# SnoRNA copy regulation affects family size, genomic location and family abundance levels

**DOI:** 10.1186/s12864-021-07757-1

**Published:** 2021-06-05

**Authors:** Danny Bergeron, Cédric Laforest, Stacey Carpentier, Annabelle Calvé, Étienne Fafard-Couture, Gabrielle Deschamps-Francoeur, Michelle S. Scott

**Affiliations:** Département de biochimie et de génomique fonctionnelle, Faculté de médecine et des sciences de la santé, Université de Sherbrooke, Sherbrooke, Québec J1E 4K8 Canada

**Keywords:** SnoRNAs, Gene expression regulation, Recombination, Retrotransposition, Gene evolution, Gene duplication, RNA-seq, Host gene, Tissue-specific regulation

## Abstract

**Background:**

Small nucleolar RNAs (snoRNAs) are an abundant class of noncoding RNAs present in all eukaryotes and best known for their involvement in ribosome biogenesis. In mammalian genomes, many snoRNAs exist in multiple copies, resulting from recombination and retrotransposition from an ancestral snoRNA. To gain insight into snoRNA copy regulation, we used Rfam classification and normal human tissue expression datasets generated using low structure bias RNA-seq to characterize snoRNA families.

**Results:**

We found that although box H/ACA families are on average larger than box C/D families, the number of expressed members is similar for both types. Family members can cover a wide range of average abundance values, but importantly, expression variability of individual members of a family is preferred over the total variability of the family, especially for box H/ACA snoRNAs, suggesting that while members are likely differentially regulated, mechanisms exist to ensure uniformity of the total family abundance across tissues. Box C/D snoRNA family members are mostly embedded in the same host gene while box H/ACA family members tend to be encoded in more than one different host, supporting a model in which box C/D snoRNA duplication occurred mostly by cis recombination while box H/ACA snoRNA families have gained copy members through retrotransposition. And unexpectedly, snoRNAs encoded in the same host gene can be regulated independently, as some snoRNAs within the same family vary in abundance in a divergent way between tissues.

**Conclusions:**

SnoRNA copy regulation affects family sizes, genomic location of the members and controls simultaneously member and total family abundance to respond to the needs of individual tissues.

**Supplementary Information:**

The online version contains supplementary material available at 10.1186/s12864-021-07757-1.

## Background

**Fig. 1:**
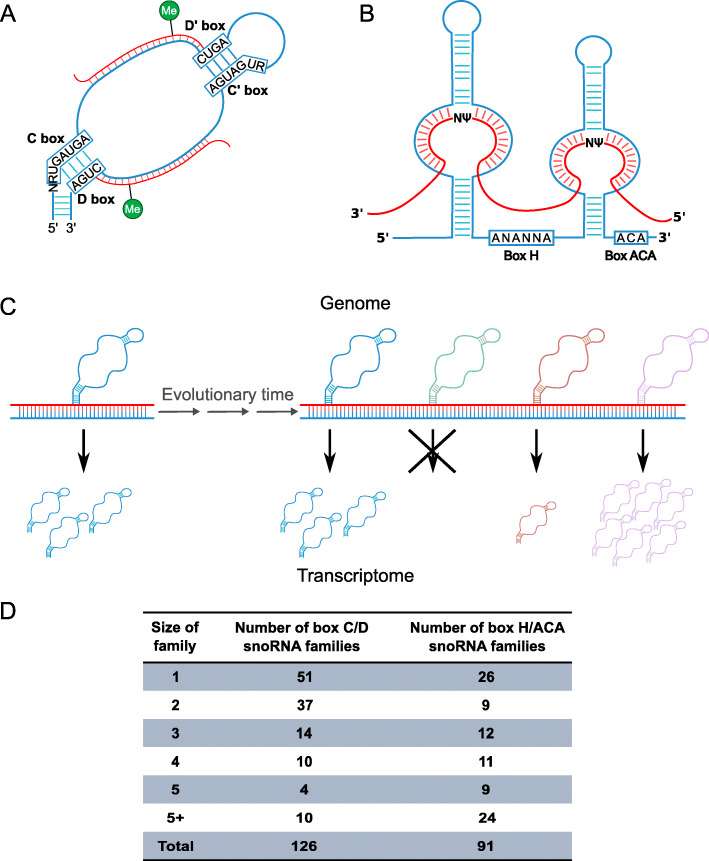
SnoRNAs exist as two main classes, each of which can be further subclassified into families. **A** Box C/D snoRNAs are characterized by the presence of boxes C and D found respectively near their 5′ and 3′ termini and interacting through non-canonical base pairing forming a k-turn. Additional boxes C′ and D′ can be found in the middle of the molecule. The antisense element, or guide region, base pairing with the target and specifying the residue to be methylated (Me) is found immediately upstream from the boxes D′ and/or D. **B** Box H/ACA snoRNAs consist of two hairpins separated by a box H (where N represents any nucleotide) and terminated by a box ACA found 3 nucleotides before the 3′ end of the molecule. The guide regions, specifying the position in the target to be pseudouridylated (Ψ) are found in bulges in the hairpins. **C** As genomes evolve over time, sequence duplication of snoRNAs, through recombination and retrotransposition mechanisms, can result in multiple copies of a parental snoRNA. Depending on the genomic context of the snoRNA copy, it can be expressed at different levels, which will likely affect the pressure under which it will be to retain its parental copy’s sequence. **D** Table indicating the number of families of different sizes for both C/D and H/ACA families in human, based on Rfam classification

Small nucleolar RNAs (snoRNAs) are a conserved, abundant and repetitive type of noncoding RNA present in all eukaryotes and a subset of archaea [1–3]. Discovered over four decades ago, snoRNAs are best characterized for their role in ribosome biogenesis, many serving as guides for the site-specific chemical modification of ribosomal RNA (rRNA) and a small number involved in rRNA processing [2, 4, 5]. Two main classes of snoRNAs have been described, differing in terms of their sequence motifs, structure, interacting proteins and, as a consequence, the chemical modification they catalyze. Box C/D snoRNAs typically range from 70 to 130 nucleotides (excluding processing snoRNAs). They are characterized by the presence of boxes C (RUGAUGA) and D (CUGA), respectively found near the 5′ and 3′ ends of the molecule and interacting through non-canonical base pairing forming a kink-turn (Fig. 1A) [6–9]. The enzymatic moiety of the box C/D snoRNA ribonucleoprotein complex (snoRNP) is the methyltransferase fibrillarin, which catalyzes 2′-O-ribose methylation of the target [1, 5, 10]. Additional boxes, C′ and D′, with same consensus sequences as boxes C and D respectively, but often less well conserved are found in the middle of the molecule [5, 9]. Box C/D snoRNAs identify their targets using their antisense element, a stretch of 10–20 nucleotides immediately upstream from the box D or D′ with strong complementarity to the target (Fig. 1A) [5, 9]. In contrast to box C/D snoRNAs, box H/ACA snoRNAs are longer (typically between 110 and 145 nucleotides), and consist of two hairpins separated by a hinge or H box (ANANNA where N can be any nucleotide) and terminated by an ACA box found 3 nucleotides from the 3′ end of the molecule (Fig. 1B) [5, 9, 11]. Box H/ACA snoRNAs interact with four conserved proteins including the pseudouridine transferase dyskerin, which catalyzes the modification of the target [12]. Box H/ACA snoRNA antisense regions with complementarity to the target are bipartite and are located in bulges in the hairpins, specifying the exact uridine to be pseudouridylated [5, 9]. In human, accepted nomenclature for snoRNA gene names starts by SNORD and SNORA for box C/D and H/ACA snoRNAs respectively [13, 14].

In addition to their role in rRNA biogenesis, a subset of snoRNAs is known to guide the modification of small nuclear RNAs (snRNAs) and the remaining snoRNAs are referred to as orphan snoRNAs [1, 15]. Over the last 15 years, however, diverse non-canonical functions for snoRNAs have been reported at several levels in the regulation of gene expression (reviewed in [4, 16, 17]). A small number of snoRNAs have been validated as carrying out both canonical and non-canonical functions [17].

In mammals, while the small number of rRNA processing snoRNAs are intergenic, expressed alone from their own promoter, almost all modification and orphan snoRNAs are encoded in introns of longer genes [2, 9, 18, 19]. Such host genes enable the expression of their encoded snoRNA, using the host promoter, in a process involving transcription, splicing, debranching and exonucleolytic degradation of the host intron. Core snoRNA binding proteins are believed to recognize and bind the snoRNA while still in its host intron, which protects the snoRNA from degradation [1, 2]. SnoRNA host genes typically either code for proteins, many of which are constituents or regulators of the ribosome or of translation, or are long noncoding RNAs (lncRNAs) [19, 20]. Human host genes can encode only one snoRNA or several snoRNAs each in their own intron [2, 19] in which case they are often copies of each other as described below. The number, genomic context and distribution of snoRNAs in genomes have been strongly shaped by evolutionary processes.

In vertebrate genomes, snoRNAs can exist in many copies thanks to mechanisms such as retrotransposition and recombination. Retrotransposons are abundant mobile genetic elements that have the capacity to copy themselves to other genomic loci through a process involving their transcription, followed by reverse transcription and insertion back into the genome [21]. However, in addition to the retrotransposons themselves, cellular RNAs such as mRNAs but also noncoding RNAs such as snoRNAs can serve as substrates for the retrotransposition machinery and be inserted into distant genomic loci, resulting in copies, in some cases numbering in the tens, hundreds and even thousands, of individual snoRNAs in genomes [3, 22–27]. SnoRNAs can also be duplicated in cis, likely through recombination, resulting in copies located in close proximity, for example in different introns of the same host gene [28, 29]. In addition to these mechanisms, copies of snoRNAs can also be generated when the host gene is duplicated, either completely or partially, also likely resulting from recombination [28]. Thus, as a consequence of retrotransposition and recombination, snoRNA copies can be inserted in a genome intronically (within the same host gene as their parental copy or in another host gene) or intergenically. If snoRNAs are inserted in a suitable location (for example with an appropriate upstream sequence to serve as a promoter, or at an appropriate position in an intron [30]), they will be expressed and could end up being important for the organism by taking over all or part of the parental copy’s functionality [31] (Fig. 1C). If the expression or lack of expression of the snoRNA is such that little selective pressure maintains its sequence, it could evolve to lose its similarity to snoRNAs. However, if expressed, this evolution could lead to the acquisition of novel interaction partners and/or targets and ultimately functions (Fig. 1C) [28, 31, 32].

Sequence identity and sequence covariance across species can be used to identify snoRNA copies and define snoRNA families. SnoRNAs with similar sequence typically are given the same name with a differing suffix (for example SNORA2A, SNORA2B and SNORA2C or SNORD1A, SNORD1B and SNORD1C). However, some snoRNAs with similar, but often not identical, sequence are given the exact same name (e.g. > 20 genes named SNORA70 in the human genome). In addition, some snoRNAs with very close sequence have different names (e.g. SNORA37 and SNORA30B; 93.8% identical). The Rfam resource provides a classification of snoRNAs across multiple organisms based on a seed alignment of curated representative sequences of each family from which a covariance model is built in an iterative manner [33]. The covariance model is then used to identify additional members from the Rfam sequence database to provide the full complement of multi member families. While the characteristic motifs and antisense elements are not explicitly taken into consideration throughout the process of building the families, these fundamental elements for canonical snoRNA functionality are expected to be highly conserved amongst family members [31, 34].

In human, the number of members per Rfam snoRNA family is variable, from 1 to over 400 members (Fig. 1D). Currently, though the duplication mechanisms to generate snoRNA families with more than one copy in a genome are generally understood, little is known of the regulation of these copies, which could offer a glimpse into their function and evolutionary pressures to maintain them. Studies of copies of other mid-size noncoding RNA biotypes have revealed that snRNA copies switch in expression between different tissue types and during development in *Drosophila*, *Xenopus*, mouse and human [35, 36]. Likewise, copies of tRNAs also display differential expression across *Caenorhabditis elegans* tissues [37]. These studies suggest that the relative expression of mid-size noncoding RNA copies is under tight regulation and that these copies could be used differentially by the cell.

To better understand human snoRNA family copy regulation, we used Rfam classification and low structure bias RNA-seq datasets across a panel of diverse normal human tissues, to characterize and compare human snoRNA family members and their abundance. We found that snoRNA families contain a variable number of members that cover a wide range of abundance. This level of abundance for each snoRNA is, to some extent, related to the level of conservation of its sequence. Furthermore, expression variability of individual members of a family is preferred over the total variability of the family, which leads to switches between most expressed members of a family across tissues. Interestingly, most of these switches include independently regulated snoRNAs encoded in the same host gene. We also found striking and subtle differences between box C/D and box H/ACA snoRNA families regarding, amongst others, the number of family members, their genomic location and their conservation, which led us to hypothesize that box C/D and box H/ACA families have evolved in a different manner.

## Results

### SnoRNA families typically consist of both highly and lowly expressed copies

**Fig. 2:**
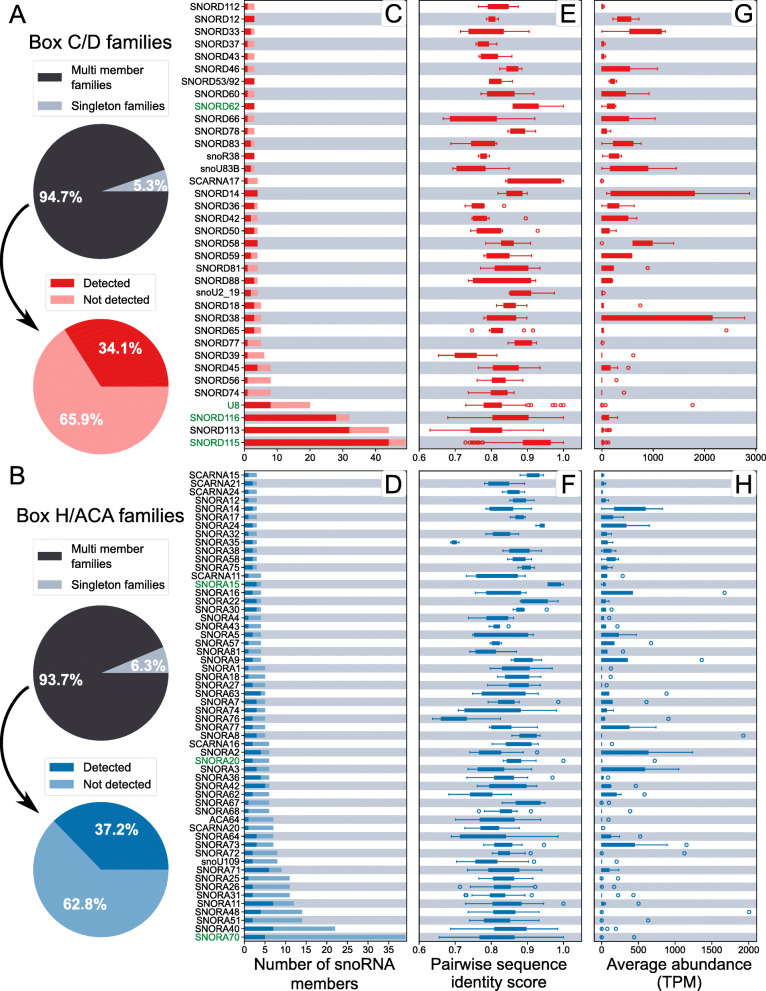
Members of most snoRNA families cover a large range of abundance values. **A**, **B** The majority of annotated human snoRNAs are members of Rfam families consisting of at least two human members (labelled as ‘Multi member families’) as shown using pie charts for box C/D (**A**, top panel) or H/ACA (**B**, top panel) snoRNAs. Families can have up to dozens of members in human, but most members have an abundance below the detectable limit (‘Not detected’, panels A and B, bottom pie charts). **C**, **D** Stacked bar charts showing the number of members for box C/D (**C**) and box H/ACA (**D**) families. Only families with at least 3 and less than 100 members are shown (for full graphs, see Figs. S1, S2). Members with abundance greater than 1 TPM in at least 1 tissue sample considered are indicated in a darker shade while non-detected members are indicated in a lighter shade. Green family names represent families with at least two identical members. **E**, **F** Family members display high levels of pairwise sequence identity. Boxplots measuring the level of pairwise sequence identity between all members of indicated families, for box C/D families in red (**E**) and H/ACA families in blue (**F**). **G**, **H** For many families, the abundance of members spans at least 3 orders of magnitude. Box plots showing the distribution of average abundance across all tissues considered of members of the indicated C/D (**G**) and H/ACA (**H**) families

To characterize human snoRNA family copy regulation, we obtained all snoRNA families with human members as defined by Rfam [33]. As indicated in Fig. 1D, this dataset consists of 126 box C/D families (totalling 967 snoRNA members) and 91 box H/ACA families (with a total of 410 snoRNA members) (Table S1). Overall, 94.7 and 93.7% of human box C/D and H/ACA snoRNAs respectively belong to an Rfam family with at least 2 human members (denoted here as multi member families, as opposed to singletons) (Fig. 2A,B, Table S2). Amongst C/D families, 75 have at least 2 human members and the number of members reaches as high as 442 for the snoU13 family (Table S2). In the case of H/ACA families, 65 have at least 2 human members and the largest family (SNORA70) has 39 members (Table S2). However, it is known that not all snoRNA genes annotated in genomes are expressed [19, 38] and non-expressed copies will not contribute to a family’s functional output. We thus took into consideration the level of expression of snoRNA family members.

To accurately quantify human snoRNA family member abundance, we took two important steps: we only considered TGIRT-seq datasets and we employed an iterative alignment strategy. TGIRT-seq is an RNA-seq approach that uses thermostable group II intron reverse transcriptases (TGIRT) to prepare the sequencing libraries. The higher fidelity and processivity of the TGIRTs compared to standard viral reverse transcriptases and the higher temperature of the reaction considerably increase the accuracy of the quantification of structured and modified RNAs such as transfer RNAs and snoRNAs [39–42]. To maximize the likelihood of aligning the reads to the right family member, we started by only accepting perfect read alignment to the human genome (no mismatches). The unaligned reads were then iteratively re-aligned to the genome accepting increasing numbers of mismatches. However, all reads aligning to snoRNAs were aligned after the second iteration (so all reads aligning to snoRNAs have at most one mismatch with the genome). Most reads (99.7%) align with no mismatches. Following this iterative alignment, aligned reads were assigned to annotated genes using CoCo which accurately attributes reads to embedded genes and distributes multimapped reads between copies proportionally to their uniquely mapped counts, important features for snoRNAs [43]. This strategy maximizes the likelihood of appropriately assigning reads to snoRNAs. Despite these precautions, it remains more difficult to ensure accurate distribution of the reads between the copies in the case of completely identical snoRNAs. Fortunately, while members of the same family can be completely identical, only 13/217 families (8 box C/D and 5 box H/ACA) analysed in this study had at least one identical pair of snoRNAs. Those families are indicated in green in all relevant figures (Fig. 2, S1, S2).

Quantification of snoRNAs from ribodepleted total RNA isolated from biological triplicates of seven normal human tissues (datasets from brain, liver, prostate, breast, ovary, testis and skeletal muscle taken from accession numbers GSE126797 and GSE157846 [19] of the Gene Expression Omnibus (GEO) repository) indicates that only 37.3 and 41.0% of C/D and H/ACA snoRNAs considered are expressed in at least one tissue, using 1 TPM in at least one sample as a definition of expression. While almost all singletons are expressed (96% for both box C/D and H/ACA), the percentage of expressed snoRNAs is even lower, 34.1 and 37.2%, respectively for box C/D and box H/ACA snoRNAs, if only multi member families are considered (Fig. 2A,B). Such low proportions of expressed annotated snoRNAs have already been noted elsewhere [19, 38]. Only 23 C/D and 2 H/ACA families with at least 2 members express all their members. Most C/D families (77.3%; 58/75) express at least half of their members but only 49.2% (32/65) H/ACA families do (Fig. 2C,D, S1, S2; for the sake of readability, only families having between 3 and 50 members are shown in Fig. 2C-H; Fig. S1 and S2 provide the full complement of families). Strikingly though, only 3 box C/D and 1 box H/ACA families have no expressed member. Interestingly, although there are more C/D than H/ACA families, H/ACA families tend to be larger, with the exception of a small number of very large C/D families, but the overall distribution of the number of expressed members is similar for both classes of snoRNAs (Fig. S3). The range of the number of expressed members per family is narrow and is centered on 2 members for both C/D and H/ACA snoRNAs (Fig. S3B).

The distribution of pairwise sequence identity between family members ranges from 66.5 to 100% for both C/D and H/ACA families (Fig. 2E,F) with averages of 81.7 and 83.7% respectively. Although all but 6 C/D and 9 H/ACA families with at least 3 members express at least one member above an average of 100 TPM across the 7 tissues considered, most families display a wide range of average abundance values across their members (Fig. 2G,H). In fact, 19/38 C/D families and 18/56 H/ACA families with at least 3 members cover at least 3 orders of magnitude for the abundance of their members (Fig. 2G,H). We found no correlation between pairwise sequence identity of a member with all other family members and its expression level (Fig. S4).

Overall, these findings reveal an evolutionary process where many snoRNAs were duplicated, giving rise to a large number of unexpressed snoRNA sequences and a smaller number of unevenly expressed snoRNA genes in the human genome. The fact that more than 98% of snoRNA families include at least one expressed member indicates that each family is distinct and is important for the cell.

### Strongly expressed family members are highly conserved across vertebrates

**Fig. 3:**
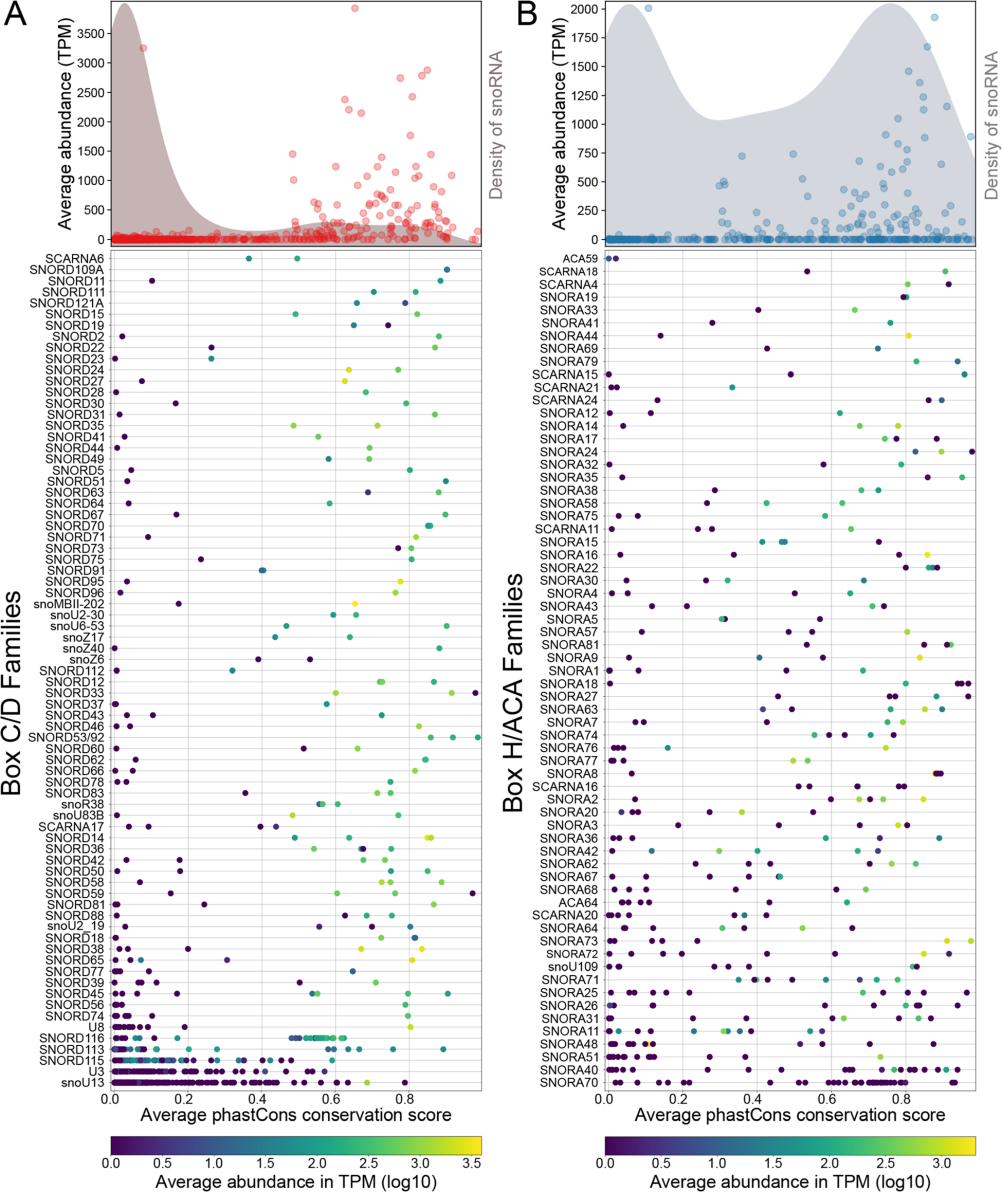
Most strongly expressed snoRNA family members in human are highly conserved throughout vertebrates. **A**, **B** Scatterplots displaying the average conservation values over the length of the snoRNA as determined using the phastCons algorithm for 100 vertebrates for all members of indicated C/D (**A**) and H/ACA (**B**) families. The color of the circles indicates the average abundance (in log10 TPM) of the family member across all human tissues considered (bottom panel). The color legend of abundance is given at the bottom of the figure. The top panel for both A and B represents scatterplots of the mean abundance in TPM of all members at a given conservation score in the panel below. The background shade shows the density of snoRNAs with a specific average phastCons conservation score

To better understand the large range of abundance values within many families, we investigated whether conservation levels correlate with expression. For both C/D and H/ACA families, members with low abundance are generally poorly conserved across vertebrates while members with high abundance are more likely to be strongly conserved across vertebrates (Fig. 3A,B). This is particularly true for box C/D snoRNAs, the vast majority of which (shaded grey distribution) are poorly conserved and only snoRNAs with a conservation score above 0.5 have the potential to be highly expressed (Fig. 3A top panel). In the case of H/ACA snoRNAs, to be highly expressed, the threshold seems lower, above a conservation score of 0.3, and there is a greater proportion of highly conserved/poorly expressed snoRNAs (Fig. 3B top panel). Moreover, the distribution of conservation for singleton families revealed that snoRNA families with only one member are highly conserved as opposed to multi member families (Fig. S5 compare C and D). Exceptions to the highly expressed/highly conserved trend could prove interesting as they might be snoRNAs acquiring new functionality. A similar although less pronounced trend is seen when considering the number of single nucleotide polymorphisms (SNPs) that exist in the human population and that are present within family members. Indeed members with lower conservation across vertebrates are more likely to harbor larger numbers of SNPs (Fig. S6, S7).

Taken together, these results suggest a selective pressure to keep the sequences of highly expressed snoRNAs unchanged. Nonetheless, this evolutionary conservation process seems to be slightly different for box C/D and box H/ACA snoRNAs, as the latter have more highly expressed/poorly conserved and poorly expressed/highly conserved members.

We also considered the distance between intronic snoRNAs and their closest downstream exon. It has previously been reported that the position for optimal expression of box C/D snoRNAs is approximately 70 nucleotides upstream of the 3′ splice site of their intron [30, 44]. The comparison of the abundance of snoRNA family members and their distance to the closest downstream exon indicates that most box C/D snoRNAs with highest abundance are indeed found within 100 nt of the 3′ splice site of their intron, although a smaller number are found further upstream (Fig. S8). In contrast, the trend is less clear for H/ACA snoRNAs (Fig. S9). It will be important to investigate more extensively the set of determinants of snoRNA expression in future analyses.

### SnoRNA expression strategies ensure low family abundance variability

**Fig. 4:**
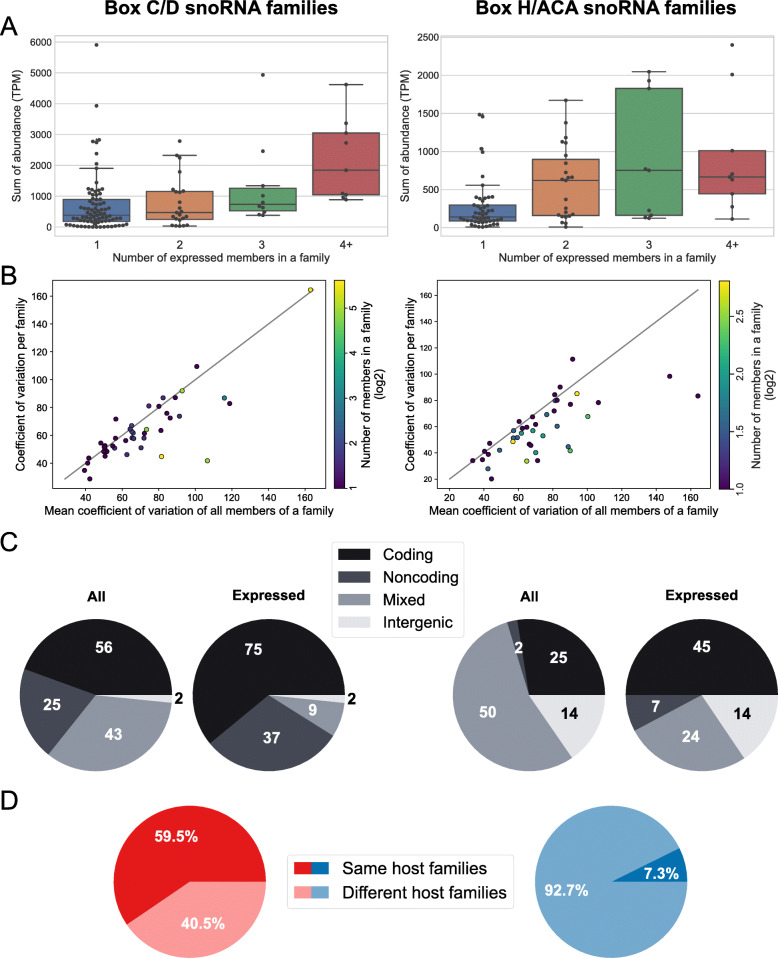
SnoRNA expression strategies ensure low family abundance variability. **A** Total family abundance tends to vary according to family size. Boxplots showing the total abundance of a family (in TPM) as a function of the number of expressed family members. **B** The average variability in abundance of individual members is greater than the variability of the family. Scatter plots showing the coefficient of variation of the total abundance of a family across human tissues as a function of the mean coefficient of variation of abundance of all members of the family. Only expressed family members are considered. The families were colored according to their numbers of expressed members. **C** For most human snoRNA families, members are intronic. As shown with pie charts, while few families (2 for box C/D families and 14 for box H/ACA families) have only intergenic members, most C/D families 64% (81/126) have only intronic members encoded in only coding genes or only noncoding genes. In contrast 55% (50/91) of H/ACA families have members encoded in different genomic context (most of these families with members both in coding and noncoding host genes, i.e. Mixed). The number of families with all their members being only in coding or noncoding genes is also highly increased if only expressed members are considered (compare Expressed vs All). Details are available in Supp Fig. S12, S13. **D** Different family member expression strategies for C/D and H/ACA families. Pie charts showing the proportion of families with all expressed members encoded in the same host gene or with some expressed members encoded in different host genes. Only families with at least two expressed members were considered. All panels of this figure show C/D families on the left and H/ACA families on the right

Variability in the number of members per family could be used by the cell to ensure sufficient total output for the family. Indeed, a weak but significant trend is observed between the number of expressed members per family and the sum of abundance of all family members (*r* = 0.23, *p*-value = 0.011 and *r* = 0.35, *p*-value = 0.0009 respectively for box C/D and box H/ACA families; Fig. 4A). Interestingly, the variability of total abundance of a family across tissues is low for all families and varies little between families for both C/D and H/ACA families, while the mean variability of individual family members is higher, with larger families generally showing larger average variability per member (Fig. 4B). This is also seen when tissues are considered individually (Fig. S10). This indicates that the cellular regulation of snoRNAs generally aims at maintaining low variability of the family abundance rather than of the abundance of individual members, as seen by most families being below the diagonal in Fig. 4B and S10. The difference between family variability and member variability is more pronounced for H/ACA snoRNAs, which show an even larger member variability for a given family variability (Fig. S11, *p*-value = 0.042 for Mann-Whitney U test), although the family size distribution of expressed members is similar between the two classes (Fig. S3B).

The genomic context of snoRNAs is an important determinant of their expression and could provide a means for the cell to control family and member abundance. To investigate the mechanisms used by the cell to control family and member abundance, we considered where snoRNAs are encoded in the genome. Most C/D families have their expressed members either all encoded in protein coding host genes or all encoded in noncoding host genes (Fig. 4C, S12, S13). In contrast, most H/ACA families have their expressed members either all encoded in protein coding host genes or encoded in both coding and noncoding host genes (ie mixed, Fig. 4C, S12, S13). In line with the differences between C/D and H/ACA families, most (59.5%) C/D families encode all their expressed members in the same host gene while only a small minority (7.3%) of H/ACA families do, most encoding their expressed members in at least two different host genes (Fig. 4D). We thus conclude that C/D and H/ACA families use different strategies to manage the abundance of their members and as a consequence, they achieve different levels of variability in family member abundance.

### Family member abundance rank can switch between tissues

**Fig. 5:**
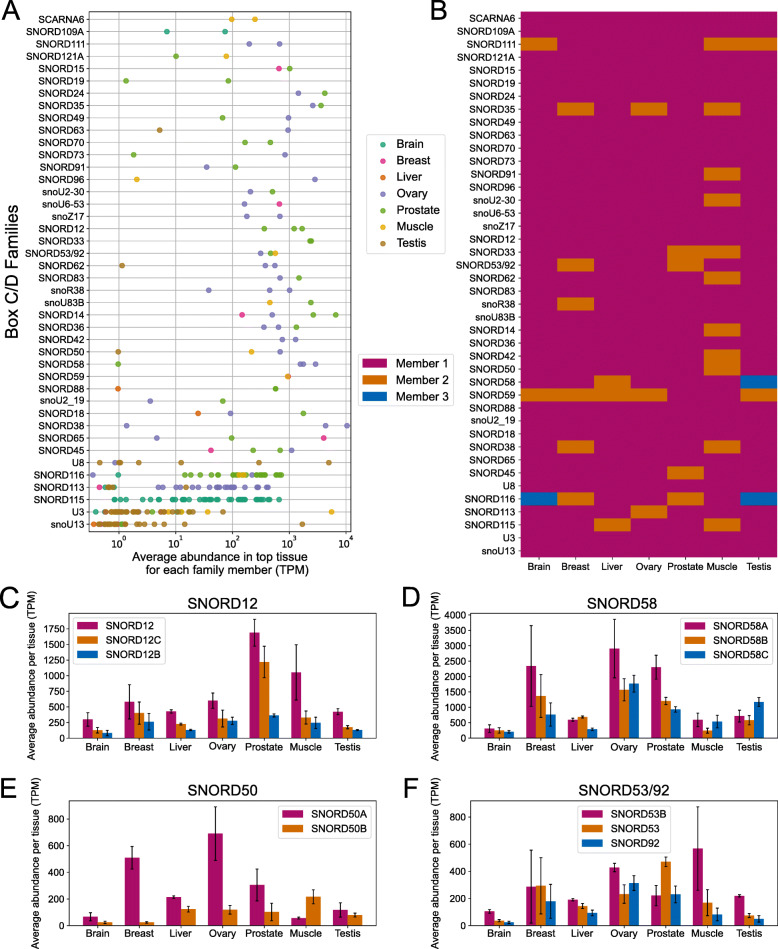
Copies can be regulated in a tissue-specific manner. **A** Differential top tissue for expressed members of a same family. Local scatterplot of each family displaying the abundance of each member of the indicated C/D families. The color of the circles represents the tissue in which the member is most abundant. **B** The rank of abundance of members of a family can change between tissues. Categorical heatmap showing the member of highest abundance for all C/D families across tissues. Only families with two or more expressed members in at least one tissue are shown. Member 1 is the member with highest total abundance in all tissues, member 2 has the second highest total abundance, and so on. Families in which at least two distinct members are the most abundant in different tissues are said to have a switch in the most abundant member across tissues. **C**-**F** Abundance patterns of family members across tissues for chosen families. Bar charts displaying the abundance of all expressed (> = 1 TPM on average) members across all tissues considered for a given family. Families can display consistent ranking across tissues as shown for the SNORD12 family, or switches between family members as shown for the SNORD58, SNORD50 and SNORD53/92 families

While total family abundance displays low variability across tissues, the variability of member abundance within families can lead to considerable differences in expression patterns across tissues. Indeed, only a minority of families with at least 2 expressed members (12/42 for C/D and 6/41 for H/ACA) have all their members most expressed in the same tissue (Fig. 5A, S14A). For example, all 3 members of the SNORD38 family are most expressed in ovary (Fig. 5A), supporting a study reporting high expression of this family in ovary of other mammals [45]. Similarly, all expressed SNORD115 members are most expressed in brain, which has been well characterized for this family [46]. In contrast, many families display variability in the top tissue for expression of their members (Fig. 5A, S14A). For example, the SNORA11 family expresses 7 members the abundance of which is highest in 3 different tissues: 4 are most expressed in ovary, 2 in testis and 1 in breast (Fig. S14A). More variability is observed in the top expression tissue of members of H/ACA families than C/D families (compare Fig. S14A and Fig. 5A). In line with the variability seen at the level of individual members for most families, tissues can display switches within families in their most abundant member. 18 C/D families and 9 H/ACA families display such switches and up to three different members of a same family can rank first in abundance when different tissues are considered (Fig. 5B, S12, S13, S14). Strikingly, 9 box C/D and 2 box H/ACA families with abundance switches between tissues have all their expressed members encoded in the same gene (Table S2).

Individual family analysis reveals different types of profiles. For example, in the SNORD12 family, which expresses three members all encoded in the same host gene, the expression of each snoRNA follows the same rank regardless of the tissue (Fig. 5C). At the opposite, in the SNORD58 family, where the three most expressed members are all embedded in the same ribosomal protein coding gene RPL17, SNORD58A is the most expressed member in brain, breast, ovary, prostate and skeletal muscle while SNORD58B is the most expressed in liver and SNORD58C is the most expressed in testis (Fig. 5D). Interestingly, in the SNORD50 family, where both expressed members are also encoded in the same lncRNA host gene, SNORD50A is the most expressed member in all tissues except in skeletal muscle (Muscle) where SNORD50B is more abundant (Fig. 5E). In the SNORD53/92 family, the rank of abundance of the three expressed members, all encoded in the same host gene, varies across tissues with SNORD53B most expressed and SNORD92 least expressed in muscle, liver, testis and brain, SNORD53 most expressed in prostate, SNORD53 and SNORD53B equally expressed in breast and SNORD53B most expressed with SNORD53 least expressed in ovary (Fig. 5F). Similar profiles can also be found in box H/ACA families (Fig. S14).

These examples suggest strong levels of regulation of individual copies. While 9 out of 18 C/D families with such switches in their most abundant member across tissues have all their expressed members encoded in the same host gene, only 2 out of 9 such H/ACA families are in this situation (Fig. S12, S13), reflecting once again the different expression strategies between the snoRNA classes. Overall, these results support the fact that members of a same family can be regulated independently whether they are in the same host gene or not.

## Discussion

SnoRNAs have long been known as multicopied genes, some existing as very large families [15, 24, 26, 46, 47]. Although the main snoRNA families have been identified [33] and the mechanisms responsible for the existence of these families such as retrotransposition and recombination have been described (e.g. [3, 22, 24, 27, 28, 48]), the regulation of the expression of snoRNA families had not been investigated systematically previously, for the whole snoRNome, in part due to a lack of suitable tools for accurate snoRNA abundance quantification. Here, using TGIRT-seq datasets, known to accurately quantify highly structured and modified RNAs including snoRNAs within the context of the whole transcriptome [19, 39, 40, 42], we considered the abundance of all human snoRNAs labelled as members of Rfam families. The 217 annotated human snoRNA families display a wide range of sizes, from one member to several hundreds, with H/ACA families tending to be larger than C/D families (Fig. 1D, 2, S1, S2, S3). Interestingly though, the range of the number of expressed snoRNAs per family is much narrower (Fig. S3), with C/D and H/ACA snoRNAs showing very close distributions, most (95.2% of C/D families and 94.5% of H/ACA families) with between 0 and 4 expressed members. Although we have not explored the expression of snoRNAs across a large diversity of conditions and tissues, considering only triplicates (from different individuals) of 7 human tissues, this study and others report similar results regarding the low proportion of expressed snoRNAs compared to the total number of annotated members [38, 39, 49]. Such a large number of non-detected snoRNA transcripts in the human annotation (approximately 60% of annotated Rfam snoRNA members not detected as expressed) is confounding, both for small- and large-scale studies. To facilitate the continued characterization of snoRNAs, we believe an expression status for each snoRNA (as expressed in at least some tissues/conditions or never detected as expressed) would be very helpful and is now available from snoDB [18].

The size of snoRNA families is an interesting consideration. Even though there is a weak significant trend between the number of expressed members of a family and the total abundance of that family (Fig. 4A), several of the smallest families are amongst the families having the highest abundance. In addition, if we take into account all the snoRNAs (expressed and not expressed), this trend becomes non-significant. With that in mind, it seems unlikely that snoRNAs are multicopied to respond to the total requirement in modification of the large numbers of rRNA transcripts. Moreover, some of the most critical regions of rRNA (for example the region near the peptidyl transferase center) are modified by snoRNA families with only one member (e.g. SNORD52, SNORA23 and SNORA54 are highly expressed singletons that guide the modifications of positions U3904 (helix H73), U4331 (helix H82) and U4539 (helix H93) of 28S RNA) [50]. The number of family members likely depends on the transposition/recombination efficiency of the family and the requirement of the family for fine-tuned regulation as discussed below.

Increasing numbers of family members enables families to cover a wide range of abundance values, which is related to their overall conservation (Fig. 3), members with highest conservation across vertebrates displaying highest expression. In addition, the total abundance of families shows low variability across tissues but some families display higher levels of member abundance variability than total family abundance variability (Fig. 4, S10, S11). Abundance variability of family members across tissues leads to switches between the most abundant member in different tissues for a subset of families (Fig. 5, S14), the cellular consequence of which will require in depth studies of individual examples in the future. Thus comparing the abundance levels of members and families across normal human tissues, it appears that while increasing the number of members does not necessarily serve the cellular purpose of increasing the total abundance of the family, it does however increase the regulation potential, and possibly the function potential, of the family.

Switches between the most abundant members of a family have been reported for other well characterized noncoding RNA classes. A study in human of U1 snRNA copies previously thought to be pseudogenes rather found them to be fully processed transcripts differentially expressed in human embryonic stem cells and HeLa cells. Blocking specific copies using antisense oligonucleotides led to global transcriptome changes, one U1 variant found to regulate the 3′ end processing of a subset of transcripts rather than their splicing [36]. A later study of snRNA expression in development identified occurrences of isoform switching between family members in organisms as distant as *Drosophila*, *Xenopus*, mouse and human [35]. In the case of snoRNAs, guide regions are typically highly conserved across same family members but many families display a small number of residues varying across the members. Abundance switches between family members with slightly different guide sequences and thus slightly differing binding affinity for rRNA targets could contribute to fractional modifications [51–54] and ultimately ribosomes with different affinity for subsets of coding transcripts. It will thus be important to pursue more extensively this line of study.

Interestingly, the top most rhythmic transcripts in a study characterizing diurnal rhythms in transcript expression across the human dorsal and ventral striatum were found to be predominantly snoRNAs (6 snoRNAs out of 20 transcripts) [55]. And amongst the top rhythmic snoRNAs are included families with switches across the tissues we considered (3/20 (50% of the snoRNAs) top rhythmic transcripts are snoRNAs (SNORA14B, SNORD115–1 and SNORA71B) from families with switches), suggesting families with capacity for variation in the abundance of members could be used by the cell in processes including the regulation of diurnal rhythms. Interestingly, from these three snoRNAs, SNORD115 members were shown to be involved in the alternative splicing of the serotonin receptor 2C gene [56] while SNORA71B was shown to promote proliferation, migration and invasion of breast cancer cells [57]. From that perspective, it is not impossible that switches in expression of family members could be used to modulate the efficiency of non-canonical functions of snoRNAs, while maintaining canonical functions.

Unexpectedly, most family members implicated in abundance switches between tissues come from the same host gene. This indicates that there exist mechanisms that regulate independently each snoRNA embedded in a same host gene. Mechanisms which could enable differential abundance of snoRNAs encoded in the same host gene include alternative splicing, the use of alternative transcription initiation/termination sites [58], and differential interacting partners which could affect snoRNA stability.

**Fig. 6:**
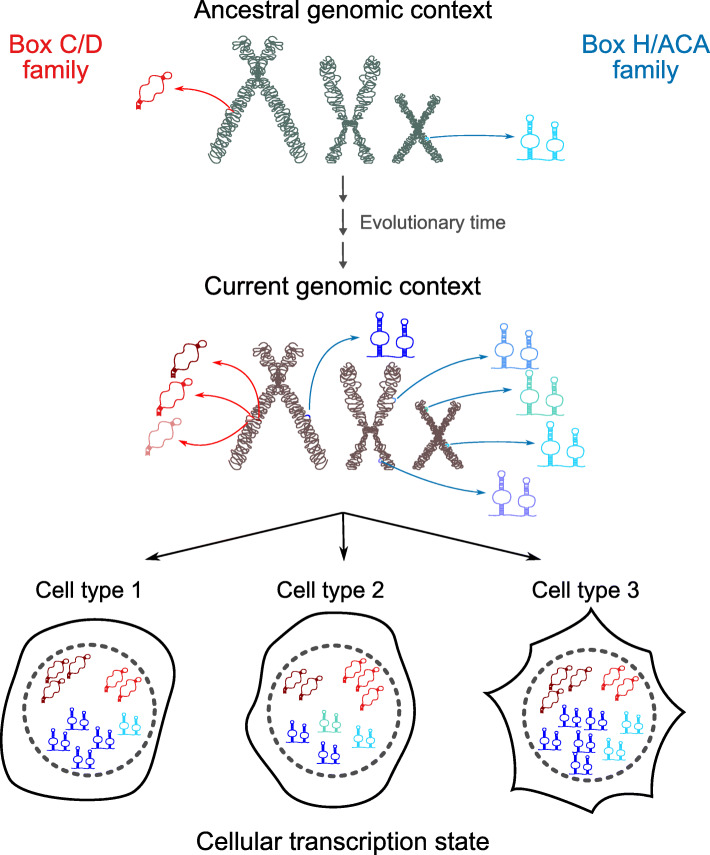
Model of differences between C/D and H/ACA families. Box C/D snoRNA families (left part) are mostly small, typically with all members encoded in the same host gene, resulting from recombination as an evolutionary force, members display low abundance variability across tissues and at the same time more switches between tissues. Box H/ACA families (right part) are larger with members encoded in more than one host gene, as a result of retrotransposition and as a consequence, members display more abundance variability across tissues

The comparison of the genomic context of C/D and H/ACA families has revealed that while C/D families typically encode all their members in the same host gene, most H/ACA families employ more than one host gene, with consequences on the variability of the members (Fig. 4, S12, S13). We thus conclude that the generation of human H/ACA copies more likely results from retrotransposition (and this evolutionary mechanism has been well characterized for H/ACAs [22, 27]) while C/D copies appear to stem mostly from recombination within the same host gene, resulting in multiple copies of the same family in different introns of the same host gene (Fig. 6). The concept that H/ACA snoRNAs are more likely than C/D snoRNAs to be captured by LINE retrotransposon machinery is supported by recent reports. Indeed, LINE-1 retrotransposition has been shown to require a 3′ poly(A) tract in both *cis* and *trans* substrates [59] and H/ACA snoRNAs, but not C/D snoRNAs have been shown in mammals to display transient 3′ poly(A) tails during their maturation [60]. These results are also consistent with a study of snoRNA copies in chicken which supports the notion that C/D snoRNAs are more likely than H/ACA snoRNAs to duplicate via cis-recombination (ie recombination within the same host gene), only 10% of chicken H/ACA snoRNAs having copies as compared to 30% of C/D snoRNAs [29]. In contrast, H/ACA copy generation is more likely to result from trans-duplication through retrotransposition, a mechanism believed to occur in mammals but not in other vertebrates [29]. Our findings in combination with diverse reported observations [22, 24, 26, 27, 29] strongly support differing mechanisms for the generation of C/D and H/ACA snoRNA copies in mammals, resulting in the differing genomic context of their family members and leading to the different characteristics for the families including as discussed above family size, variability and co-regulation of the members (Fig. 6).

## Conclusions

Our study of copy regulation of snoRNAs led us to discover that the number of expressed snoRNA family members, unlike total family members, is narrow in range with generally no more than 4 expressed members per family. Larger family size does not necessarily result in higher total family abundance, indicating that snoRNAs are not duplicated to meet a higher abundance requirement of the family in the cell. Expression variability of individual members is preferred over the total variability for families, supporting the importance of the family unit in cell function. In addition, the most expressed snoRNAs in a family can switch in abundance between tissues indicating that the cell likely uses small differences (base pair composition, structure, etc.) between family members to fine tune cellular processes, like rRNA processing or other non-canonical functions, while maintaining a more stable level of total family abundance. Importantly, the snoRNA location, generally within the same host gene for box C/D families and dispersed throughout the genome for box H/ACA families, as well as the higher number of members for H/ACA families strongly support box C/D snoRNA duplication resulting from cis-recombination and box H/ACA duplication from retrotransposition.

## Methods

### Assembling Rfam family dataset and snoRNA features

All Rfam families including at least one human snoRNA were obtained from Rfam [33] through the snoDB database [18]. The Rfam family names were used to identify the families. All other snoRNA information such as type, RNA sequence and host id and gene name was taken from the snoDB database [18].

### RNA-seq datasets and processing pipeline

TGIRT-seq datasets generated from 7 different normal human tissues (ovary, breast, prostate, prostate, skeletal muscle, liver, brain; 3 different samples from different individuals for each tissue) were obtained from a previous study [19]. The datasets are available from the Gene Expression Omnibus, under the accession numbers GSE126797 and GSE157846. As described in [19], all libraries were prepared from total RNA using ribodepletion following the TGIRT-seq protocol and were paired-end sequenced on an Illumina NextSeq500 sequencer. Datasets were then analyzed using a succession of bioinformatics tools implemented in a reproducible Snakemake workflow [61] available at http://gitlabscottgroup.med.usherbrooke.ca/lafced/Stringent_RNA_seq/tree/master. Briefly, paired-end reads were first trimmed using Trimmomatic v0.36 [62] (with the following parameters: ILLUMINACLIP:<fastaWithAdaptersEtc>:2:12:10:8, TRAILING:30, LEADING:30, MINLEN:20, all other parameters at default values) to remove adapters and low-quality reads. FastQC v0.11.5 was employed before and after trimming to assess the quality of the reads. Trimmed reads were aligned to the human genome assembly GRCh38 (hg38, v101). The alignment was done in an iterative manner using the aligner STAR v2.6.1a [63], iterating from 0 to 5 for the parameter –outFilterMismatchNmax, to ensure that the reads are aligned with the snoRNA copy to which they align best. For all iterations, the other parameters were set to: --runMode alignReads, outFilterScoreMinOverLread 0.3, −-outFilterMatchNminOverLread 0.3, −-outFilterMultimapNmax 100, −-winAnchorMultimapNmax 100, −-alignEndsProtrude 5 ConcordantPair, all other parameters at default values. The index required to align reads to the human genome was generated using STAR v2.6.1a (with the following parameters: --runMode genomeGenerate and --sjdbOverhang 74). BAM files resulting from the iterative alignment were merged using samtools v1.9 [64].

Counts were attributed to genomic features using CoCo v0.2.1p4 [43] (with the following parameters: cc --strand 1 --paired, all other parameters at default values), using our custom annotation (.gtf file available at https://zenodo.org/record/4570182/files/hg38_Ensembl_V101_Scottlab_2020.gtf). Normalized counts in transcripts per million (TPM) were obtained from the output of CoCo. Only snoRNAs with an abundance greater than 1 TPM in at least one tissue sample are referred to as “expressed” or “detected” snoRNAs.

### Pairwise sequence identity of all snoRNA within a family

Sequence identity was computed for all pairs of snoRNAs, for each family, using the Biopython pairwise2.align.globalxx function [65]. The score obtained (number of aligned residues) was divided by the shortest of the two snoRNAs to obtain what we report as the pairwise sequence identity score.

### Conservation score across vertebrates

Using a python script that considers the annotation file described above and the “phastCons 100 Vertebrates” track from the UCSC Genome Browser [66, 67], a conservation score was associated to each nucleotide of a snoRNA using bedtools (v2.26.0) intersect [68] and the conservation score per snoRNA was generated by calculating the average score of all the nucleotides included in that snoRNA sequence.

### Levels of single nucleotide polymorphisms (SNPs) in snoRNAs

The position of all human SNPs was obtained from the UCSC Genome Browser track dbSnp153Common.bb generated from SNPdb build 153 (http://hgdownload.soe.ucsc.edu/gbdb/hg38/snp/dbSnp153Common.bb) [69], which includes only common SNPs with minor allele frequency > 1%. Bedtools intersect [68] was used to associate SNPs to snoRNAs.

### Intronic snoRNA distance to the closest downstream exon of their host gene

The position of intronic snoRNAs within their host gene was computed using a python script and the annotation file described above. The transcripts per host gene were first sorted by total number of exons and by transcript name (in descending and ascending order, respectively). We then iterated through these sorted transcripts until the first non-overlapping host transcript was found and thereby assigned to the corresponding snoRNA. The snoRNA position within its host gene was thus computed as the distance between the 3′ end of the snoRNA and the start of the first downstream exon in the identified host transcript.

## Supplementary Information

**Additional file 1: Table S1.** Rfam classified snoRNAs and their characteristics. ‘Expressed’ refers to at least 1 TPM of abundance in one of the 21 samples (7 tissues). Average_TPM, Min_TPM and Max_TPM is the average, max and min values, respectively, of the 21 samples. Tissue_most_expressed_in is the tissue where the average abundance of a tissue is the highest. Conservation_score refers to the average of the “phastCons 100 Vertebrates” score for the entire snoRNA divided by the length of the sequence. Alignement_score is the average pairwise sequence identity score for a snoRNA against all the other members of a family. The coefficient of variation (CV = σ / μ * 100, σ being the standard deviation and μ the mean) was calculated from the mean value of each tissue. Genomic_context displays the location of the snoRNA, which could be either coding (in a protein coding gene), noncoding (in a noncoding gene) or intergenic. SNP/nt_1000 is the number of SNP * 1000 divided by the length of the snoRNA.

**Additional file 2: Table S2.** Rfam classified snoRNA families and their characteristics. ‘Expressed’ refers to at least one family member expressed, according to our criteria (see Table S1). Average_sum_TPM is the average of the sum of all snoRNA family members across the 21 samples. Num_members and Num_expressed_members are the number of all and expressed members of a family, respectively. Switch refers to the presence of switches between the most abundant member across tissues. Same_host displays TRUE if all expressed members are in the same host gene. Family_covariation is the covariation (see Table S1) of the sum of abundance of all members of a family across tissues. Mean_member_covariation is the mean covariation of each expressed member of a family across tissues.

**Additional file 3: Figure S1.** Number of members and abundance status for all C/D snoRNA families with at least 2 members. Stacked bar chart showing the number of members for box C/D families. Members with abundance greater than 1 TPM in at least 1 tissue considered are indicated in a darker shade while non-detected members are indicated in a lighter shade. Families with at least one identical pair of snoRNAs are indicated in green.

**Additional file 4: Figure S2.** Number of members and abundance status for all H/ACA snoRNA families with at least 2 members. Stacked bar chart showing the number of members for box H/ACA families. Members with abundance greater than 1 TPM in at least 1 tissue considered are indicated in a darker shade while non-detected members are indicated in a lighter shade. Families with at least one identical pair of snoRNAs are indicated in green.

**Additional file 5: Figure S3.** Distribution of family sizes for box C/D (red) and H/ACA (blue) snoRNAs considering all members (A) and only expressed members (B).

**Additional file 6: Figure S4.** Sequence similarity between members of their family is not correlated with expression abundance. Scatterplot displaying the average pairwise alignment score of a given member to all other members of the family for all members of C/D (A) and H/ACA (B) families. The color of the circles indicates the average abundance (in log10 TPM) of the family member across all human tissues considered (bottom panel). The color legend of abundance is given at the bottom of the figure. The top panel for both A and B represents scatterplots of the mean abundance in TPM of all members at a given pairwise identity score in the panel below.

**Additional file 7: Figure S5.** Singleton snoRNAs are more conserved than multi member family members. (A-D) Density plots showing the distribution of the number of snoRNAs with a specific average phastCons conservation for all (A), expressed (B), multi member families (C) or singleton (D) snoRNAs, for box C/D (red) or box H/ACA (blue).

**Additional file 8: Figure S6.** Box C/D family members with high conservation across vertebrates are less likely to carry polymorphisms across humans. Scatterplot displaying the average conservation values over the length of the snoRNA as determined using the phastCons algorithm for 100 vertebrates for all members of C/D families. The color of the circles indicates the number of single nucleotide polymorphisms (SNP) according to dbSNP build 153 for a given member, normalized by its length. The color legend of SNP*1000/length is given on the bottom. The top panel represents a bar chart of the mean number of SNP per snoRNA length at a given conservation score in the panel below. No correlation was found between the SNP position within the snoRNA and the snoRNA abundance.

**Additional file 9: Figure S7.** Box H/ACA family members with high conservation across vertebrates are less likely to carry polymorphisms across humans. Scatterplot displaying the average conservation values over the length of the snoRNA as determined using the phastCons algorithm for 100 vertebrates for all members of H/ACA families. The color of the circles indicates the number of single nucleotide polymorphisms (SNP) according to dbSNP build 153 for a given member, normalized for its length. The color legend of SNP*1000/length is given on the bottom. The top panel represents a bar chart of the mean number of SNP per snoRNA length at a given conservation score in the panel below. No correlation was found between the SNP position within the snoRNA and the snoRNA abundance.

**Additional file 10: Figure S8.** Most strongly expressed C/D snoRNA family members are generally closer to their downstream exon. Scatterplot displaying the absolute distance of snoRNA from the downstream exon for all members of C/D families (in log10 nt). The color of the circles indicates the average abundance (in log10 TPM) of the family member across all human tissues considered (middle panel). The top panel is a scatterplot of the mean abundance in TPM of all members at a given absolute distance from the downstream exon in the panel below. The background shade shows the density of snoRNAs with a specific absolute distance from the downstream exon. The bottom panel is a schematic representation of the variable length of the intron in which a snoRNA is located (orange solid and dotted line) and the downstream exon (rust colored rectangle).

**Additional file 11: Figure S9.** SnoRNA family member abundance as a function of their distance to the closest downstream exon. Scatterplot displaying the absolute distance of snoRNA from the downstream exon for all members of H/ACA families (in log10 nt). The color of the circles indicates the average abundance (in log10 TPM) of the family member across all human tissues considered (middle panel). The top panel is a scatterplot of the mean abundance in TPM of all members at a given absolute distance from the downstream exon in the panel below. The background shade shows the density of snoRNAs with a specific absolute distance from the downstream exon. The bottom panel is a schematic representation of the variable length of the intron in which a snoRNA is located (orange solid and dotted line) and the downstream exon (rust colored rectangle).

**Additional file 12: Figure S10.** The average variability in abundance of individual members is greater than the overall variability of the family for most tissues. Scatter plots showing the coefficient of variation of the total abundance of a family across samples of a specific tissue as a function of the mean coefficient of variation of abundance of all members of the family in the tissue. C/D snoRNA families are shown in the graphs in the left and H/ACA snoRNAs are shown on the right. Only expressed family members are considered. The families were colored according to their numbers of expressed members (legend shown at bottom).

**Additional file 13: Figure S11.** Box H/ACA families display higher member variability relative to their total family variability compared to box C/D. Density plot showing the distribution of the distance of each family from the linear function x = y, representing equal total family covariation and mean family members covariation (see Fig. 4B). Negative values represent higher mean family members covariation while positive values represent higher family covariation. Box H/ACA distribution (blue) is significantly different (shifted towards the left) from box C/D distribution (red) according to the Mann-Whitney U test (*p*-value = 0.042).

**Additional file 14: Figure S12.** Human C/D snoRNAs are mostly intronic and members are mostly encoded in only one host gene. Bar charts displaying the number of intronic (in both coding (A) and noncoding (B) host genes) or intergenic (C) members for box C/D families. Only expressed members were shown in the bar charts. In the case of intronic members, the different colors represent different host genes. The proportion of expressed and non-expressed members encoded in intronic coding, intronic noncoding and intergenic regions are shown respectively in D, E and F. The violet in snoRNA family names highlights the presence of a tissue abundance switch between members in the family.

**Additional file 15: Figure S13.** Human H/ACA snoRNAs are mostly intronic and members can be encoded in more than one distinct host gene. Bar charts displaying the number of intronic (in both coding (A) and noncoding (B) host genes) or intergenic (C) members for box H/ACA families. Only expressed members were shown in the bar charts. In the case of intronic members, the different colors represent different host genes. The proportion of expressed and non-expressed members encoded in intronic coding, intronic noncoding and intergenic regions are shown respectively in D, E and F. The violet in snoRNA family names highlights the presence of a tissue abundance switch between members in the family.

**Additional file 16: Figure S14.** H/ACA snoRNA copies can be regulated in a tissue-specific manner. (A) Differential top tissue for expressed members of a same family. Local scatterplot of each family displaying the abundance of each member of the indicated H/ACA families. The color of the circles represents the tissue in which the member is most abundant. (B) The rank of abundance of members of a family can change between tissues. Categorical heatmap showing the member of highest abundance for all H/ACA families across tissues. Only families with two or more expressed members in at least one tissue are shown. Member 1 is the member with highest total abundance in all tissues, member 2 has the second highest total abundance, and so on. Families in which at least two distinct members are the most abundant in tissues are said to have a switch in the most abundant member across tissues. (C-F) Abundance patterns of family members across tissues. Bar charts displaying the abundance of all expressed (> = 1 average TPM) members across all tissues considered for a given family. Families can display consistent ranking across tissues as shown for the SNORA7 family, or switches between family members as shown for the SNORA71, SNORA14 and SNORA77 families.

## Data Availability

The RNA-seq datasets are available from the Gene Expression Omnibus (GEO) repository, under the accession numbers GSE126797 (https://www.ncbi.nlm.nih.gov/geo/query/acc.cgi?acc=GSE126797) and GSE157846 (https://www.ncbi.nlm.nih.gov/geo/query/acc.cgi?acc=GSE157846). The custom annotation used for the analysis (.gtf file) is available in Zenodo at https://zenodo.org/record/4570182/files/hg38_Ensembl_V101_Scottlab_2020.gtf. The RNA-seq pipeline used to extract the snoRNA expression profiles is accessible from our Gitlab server at http://gitlabscottgroup.med.usherbrooke.ca/lafced/Stringent_RNA_seq/tree/master. All other data used and generated are available in the article and its supplementary tables.
