# Supplementary material for: SnoRNA copy regulation affects family size, genomic location and family abundance levels
Source: BMC Genomics. 2021 Jun 5;22:414. doi: 10.1186/s12864-021-07757-1 (PMC8178906; doi:10.1186/s12864-021-07757-1)
Supplement: Supplementary file 3 — Additional file 3: Figure S1. Number of members and abundance status for all C/D snoRNA families with at least 2 members. Stacked bar chart showing the number of members for box C/D families. Members with abundance greater than 1 TPM in at least 1 tissue considered are indicated in a darker shade while non-detected members are indicated in a lighter shade. Families with at least one identical pair of snoRNAs are indicated in green. [file 12864_2021_7757_MOESM3_ESM.pdf]

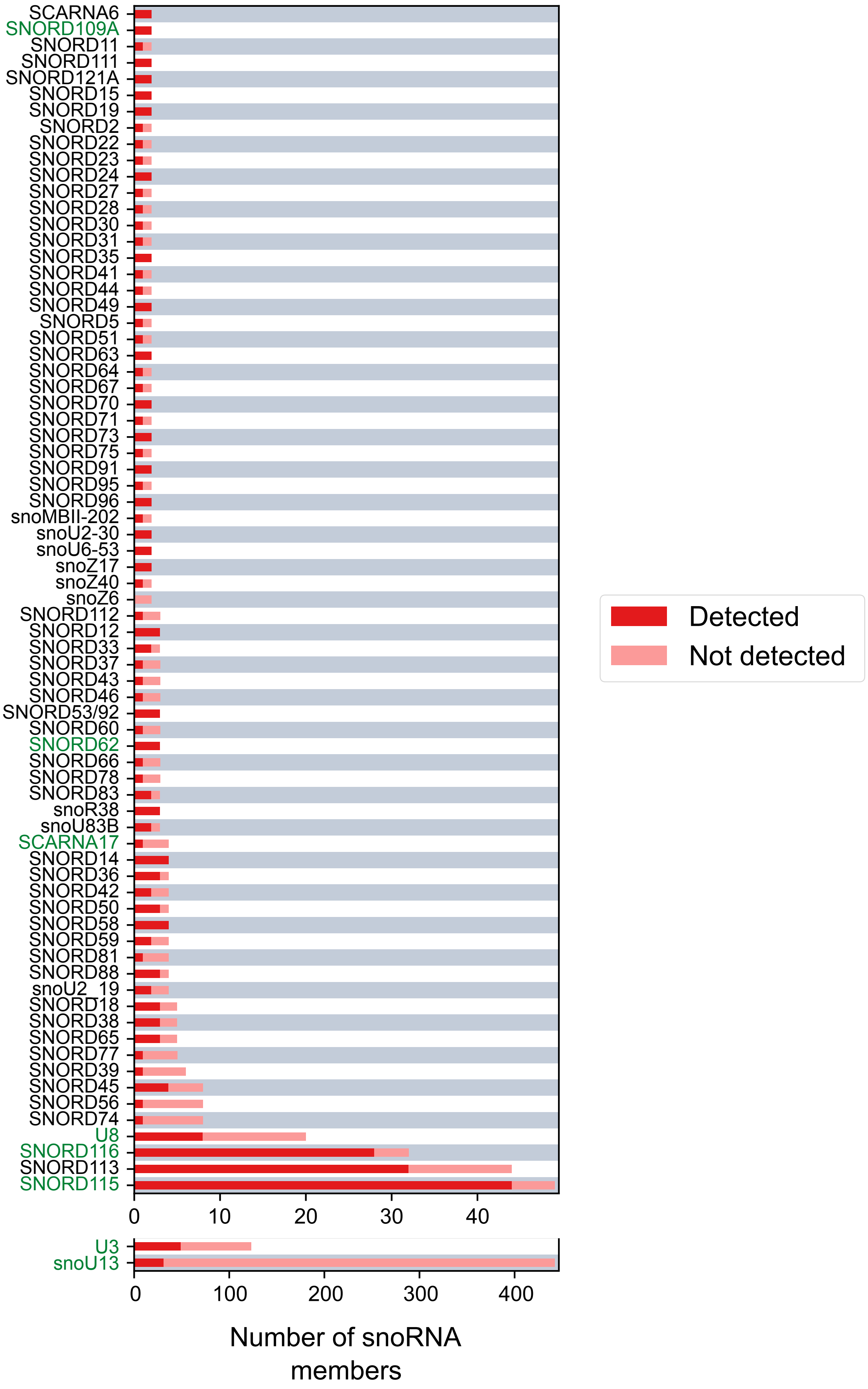

Figure S1: Number of members and abundance status for all C/D snoRNA families with at least 2 members. Stacked bar chart showing the number of members for box C/D families. Members with abundance greater than 1 TPM in at least 1 tissue considered are indicated in a darker shade while non-detected members are indicated in a lighter shade. Families with at least one identical pair of snoRNAs are indicated in green.
