# Supplementary material for: SnoRNA copy regulation affects family size, genomic location and family abundance levels
Source: BMC Genomics. 2021 Jun 5;22:414. doi: 10.1186/s12864-021-07757-1 (PMC8178906; doi:10.1186/s12864-021-07757-1)
Supplement: Supplementary file 5 — Additional file 5: Figure S3. Distribution of family sizes for box C/D (red) and H/ACA (blue) snoRNAs considering all members (A) and only expressed members (B). [file 12864_2021_7757_MOESM5_ESM.pdf]

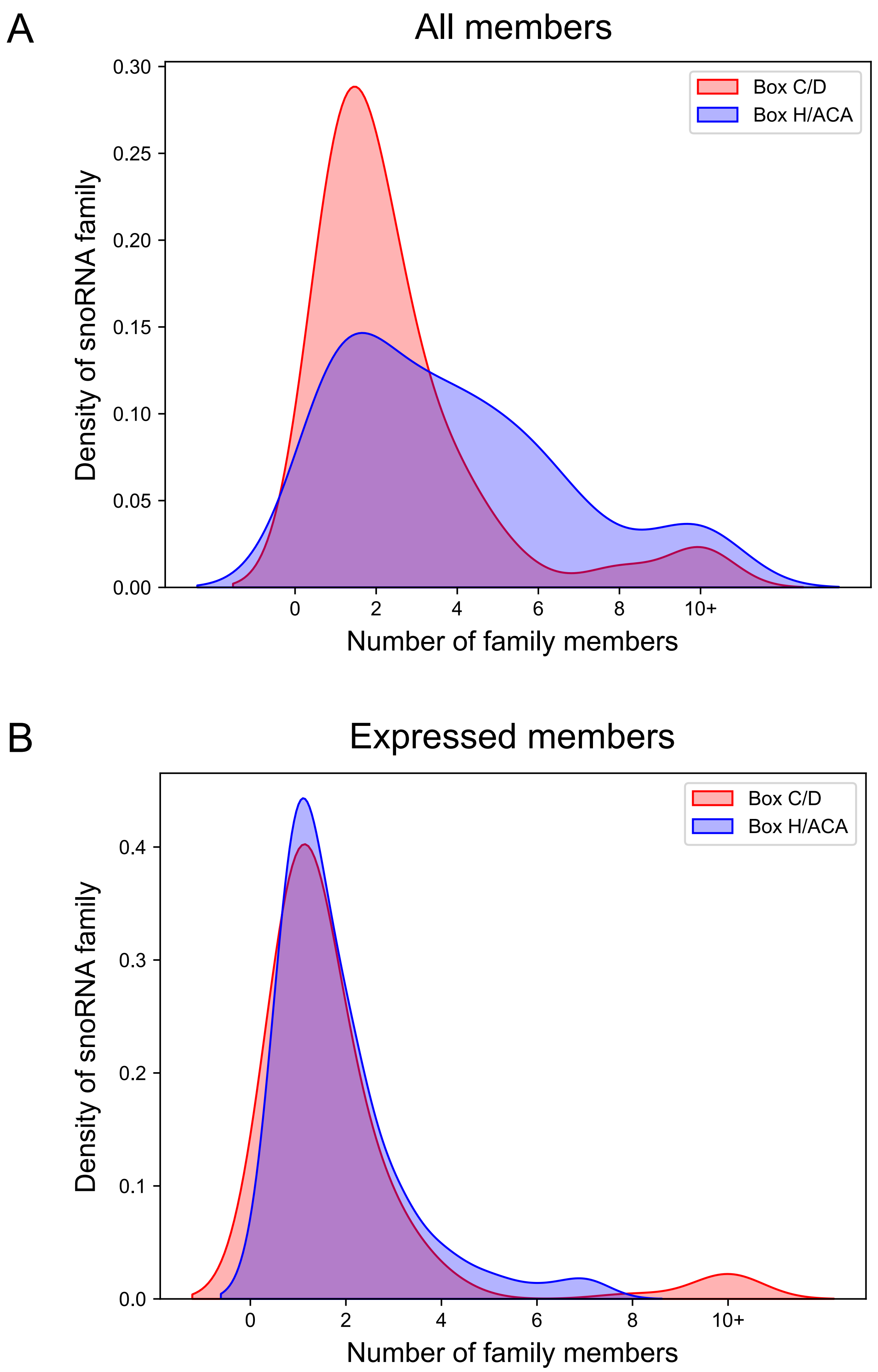

Figure S3: Distribution of family sizes for box C/D (red) and H/ACA (blue) snoRNAs considering all members (A) and only expressed members (B).
