# Supplementary material for: SnoRNA copy regulation affects family size, genomic location and family abundance levels
Source: BMC Genomics. 2021 Jun 5;22:414. doi: 10.1186/s12864-021-07757-1 (PMC8178906; doi:10.1186/s12864-021-07757-1)
Supplement: Supplementary file 6 — Additional file 6: Figure S4. Sequence similarity between members of their family is not correlated with expression abundance. Scatterplot displaying the average pairwise alignment score of a given member to all other members of the family for all members of C/D (A) and H/ACA (B) families. The color of the circles indicates the average abundance (in log10 TPM) of the family member across all human tissues considered (bottom panel). The color legend of abundance is given at the bottom of the figure. The top panel for both A and B represents scatterplots of the mean abundance in TPM of all members at a given pairwise identity score in the panel below. [file 12864_2021_7757_MOESM6_ESM.pdf]

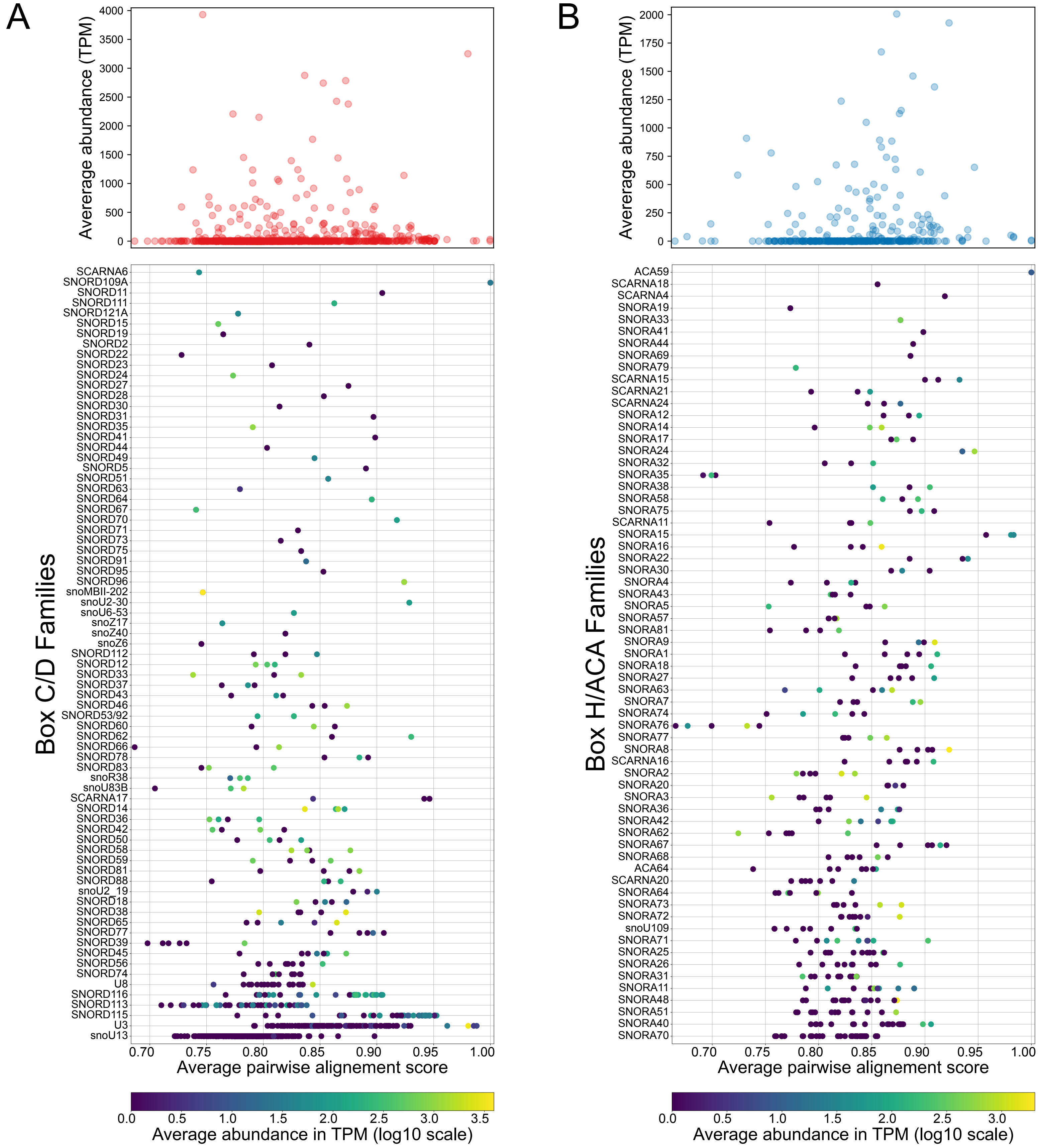

Figure S4: Sequence similarity between members of their family is not correlated with expression abundance. Scatterplot displaying the average pairwise alignment score of a given member to all other members of the family for all members of C/D (A) and H/ACA (B) families. The color of the circles indicates the average abundance (in log10 TPM) of the family member across all human tissues considered (bottom panel). The color legend of abundance is given at the bottom of the figure. The top panel for both A and B represents scatterplots of the mean abundance in TPM of all members at a given pairwise identity score in the panel below.
