# Supplementary material for: SnoRNA copy regulation affects family size, genomic location and family abundance levels
Source: BMC Genomics. 2021 Jun 5;22:414. doi: 10.1186/s12864-021-07757-1 (PMC8178906; doi:10.1186/s12864-021-07757-1)
Supplement: Supplementary file 7 — Additional file 7: Figure S5. Singleton snoRNAs are more conserved than multi member family members. (A-D) Density plots showing the distribution of the number of snoRNAs with a specific average phastCons conservation for all (A), expressed (B), multi member families (C) or singleton (D) snoRNAs, for box C/D (red) or box H/ACA (blue). [file 12864_2021_7757_MOESM7_ESM.pdf]

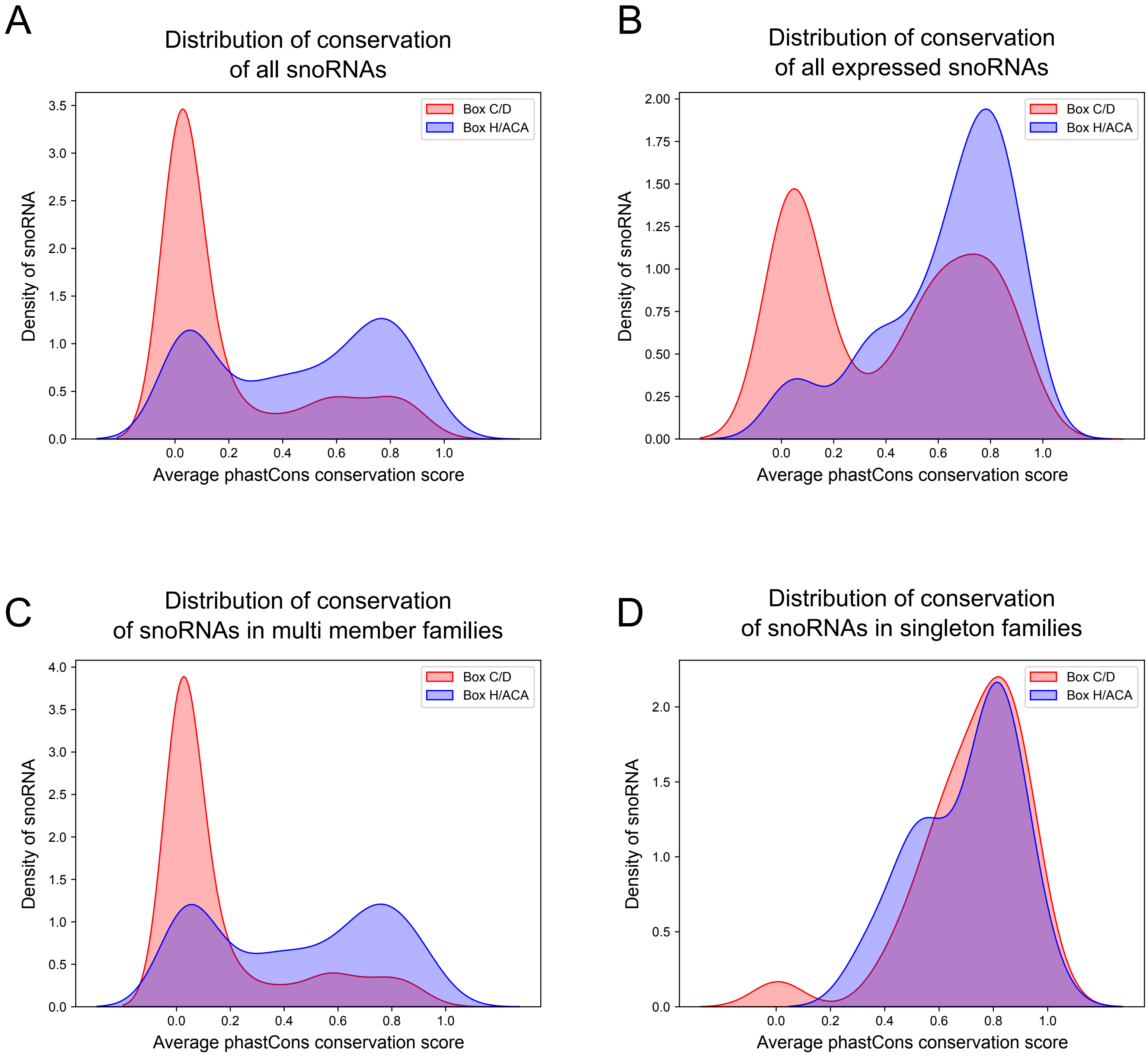

Figure S5: Singleton snoRNAs are more conserved than multi member family members. (A-D) Density plots showing the distribution of the number of snoRNAs with a specific average phastCons conservation for all (A), expressed (B), multi member families (C) or singleton (D) snoRNAs, for box C/D (red) or box H/ACA (blue).
