# Supplementary material for: SnoRNA copy regulation affects family size, genomic location and family abundance levels
Source: BMC Genomics. 2021 Jun 5;22:414. doi: 10.1186/s12864-021-07757-1 (PMC8178906; doi:10.1186/s12864-021-07757-1)
Supplement: Supplementary file 8 — Additional file 8: Figure S6. Box C/D family members with high conservation across vertebrates are less likely to carry polymorphisms across humans. Scatterplot displaying the average conservation values over the length of the snoRNA as determined using the phastCons algorithm for 100 vertebrates for all members of C/D families. The color of the circles indicates the number of single nucleotide polymorphisms (SNP) according to dbSNP build 153 for a given member, normalized by its length. The color legend of SNP*1000/length is given on the bottom. The top panel represents a bar chart of the mean number of SNP per snoRNA length at a given conservation score in the panel below. No correlation was found between the SNP position within the snoRNA and the snoRNA abundance. [file 12864_2021_7757_MOESM8_ESM.pdf]

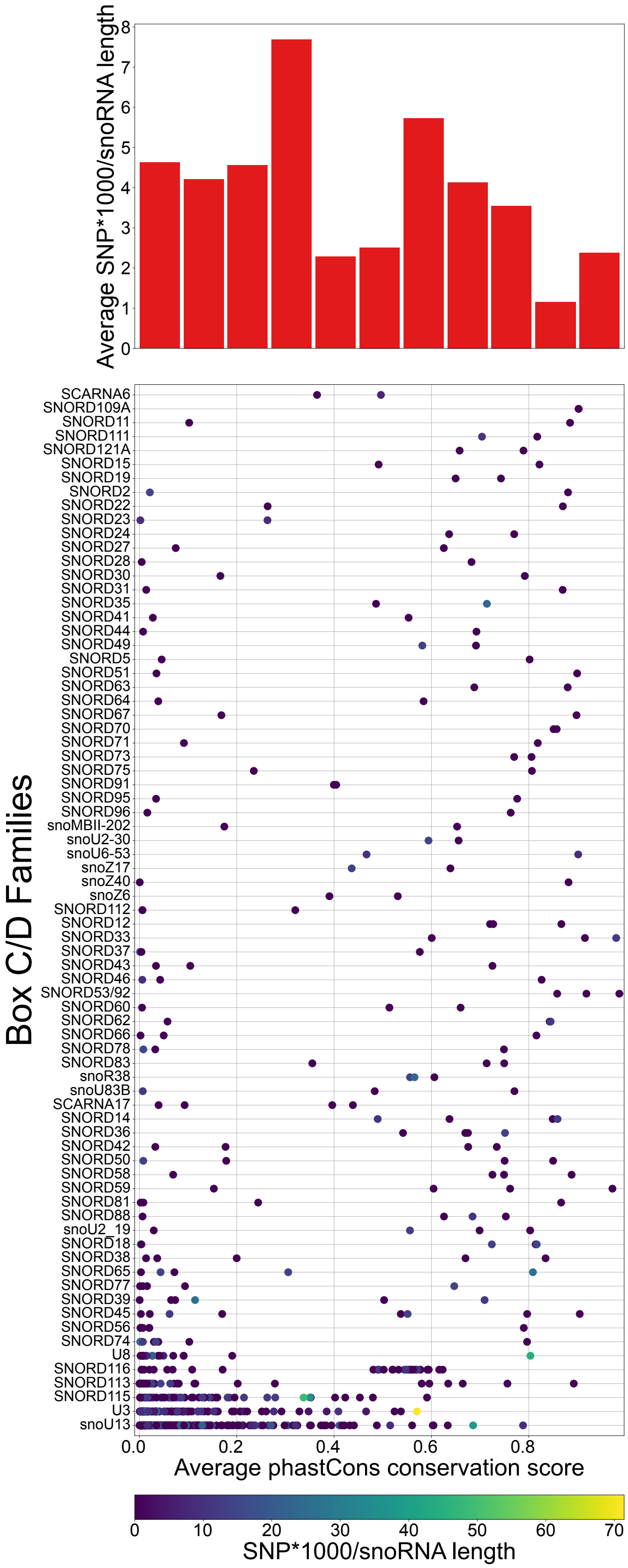


**Figure S6: Box C/D family members with high conservation across vertebrates are less likely to carry polymorphisms across humans.** Scatterplot displaying the average conservation values over the length of the snoRNA as determined using the phastCons algorithm for 100 vertebrates for all members of C/D families. The color of the circles indicates the number of single nucleotide polymorphisms (SNP) according to dbSNP build 153 for a given member, normalized by its length. The color legend of SNP*1000/length is given on the bottom. The top panel represents a bar chart of the mean number of SNP per snoRNA length at a given conservation score in the panel below. No correlation was found between the SNP position within the snoRNA and the snoRNA abundance.
