# Supplementary material for: SnoRNA copy regulation affects family size, genomic location and family abundance levels
Source: BMC Genomics. 2021 Jun 5;22:414. doi: 10.1186/s12864-021-07757-1 (PMC8178906; doi:10.1186/s12864-021-07757-1)
Supplement: Supplementary file 10 — Additional file 10: Figure S8. Most strongly expressed C/D snoRNA family members are generally closer to their downstream exon. Scatterplot displaying the absolute distance of snoRNA from the downstream exon for all members of C/D families (in log10 nt). The color of the circles indicates the average abundance (in log10 TPM) of the family member across all human tissues considered (middle panel). The top panel is a scatterplot of the mean abundance in TPM of all members at a given absolute distance from the downstream exon in the panel below. The background shade shows the density of snoRNAs with a specific absolute distance from the downstream exon. The bottom panel is a schematic representation of the variable length of the intron in which a snoRNA is located (orange solid and dotted line) and the downstream exon (rust colored rectangle). [file 12864_2021_7757_MOESM10_ESM.pdf]

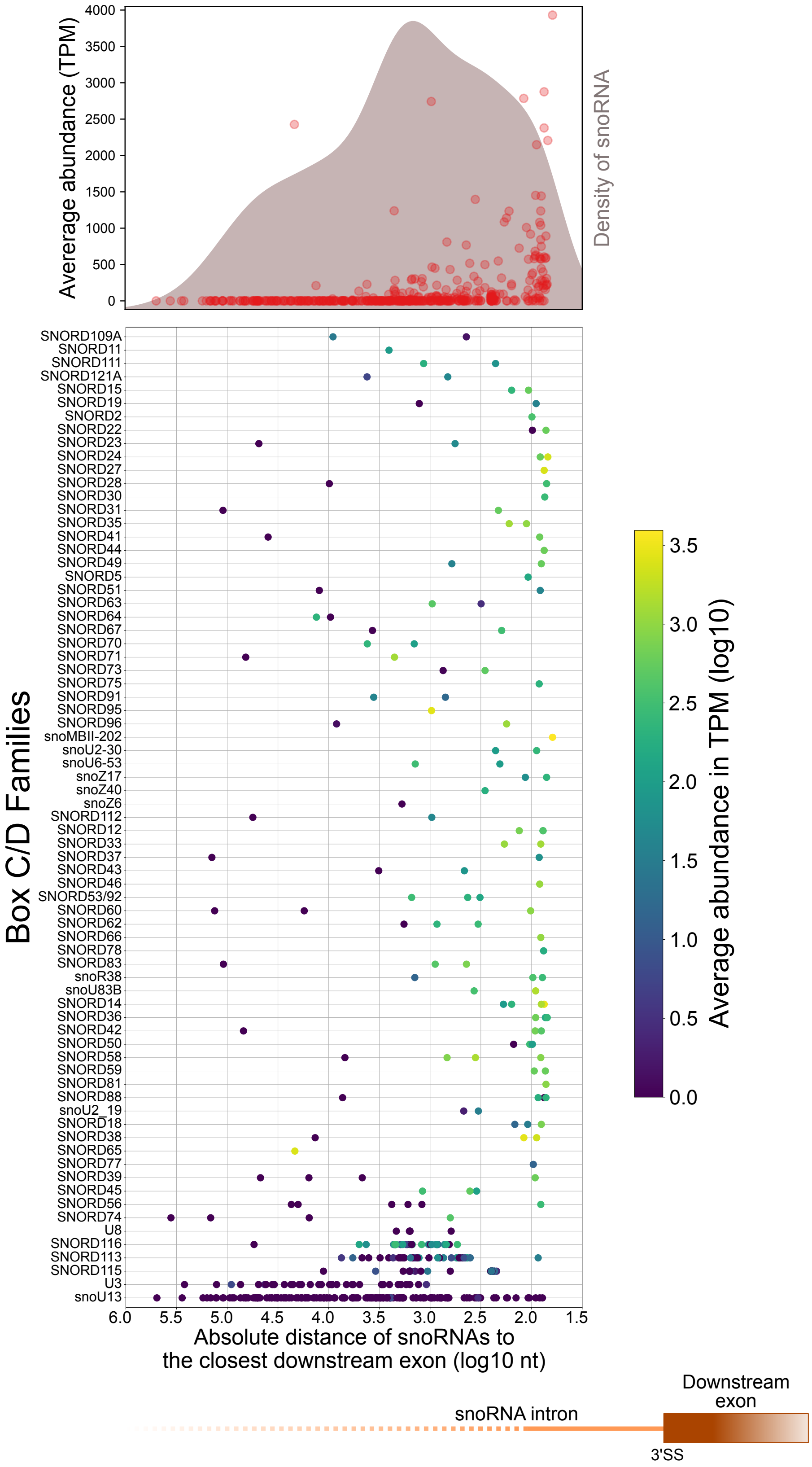

Figure S8: Most strongly expressed C/D snoRNA family members are generally closer to their downstream exon. Scatterplot displaying the absolute distance of snoRNA from the downstream exon for all members of C/D families (in log10 nt). The color of the circles indicates the average abundance (in log10 TPM) of the family member across all human tissues considered (middle panel). The top panel is a scatterplot of the mean abundance in TPM of all members at a given absolute distance from the downstream exon in the panel below. The background shade shows the density of snoRNAs with a specific absolute distance from the downstream exon. The bottom panel is a schematic representation of the variable length of the intron in which a snoRNA is located (orange solid and dotted line) and the downstream exon (rust colored rectangle).
