# Supplementary material for: SnoRNA copy regulation affects family size, genomic location and family abundance levels
Source: BMC Genomics. 2021 Jun 5;22:414. doi: 10.1186/s12864-021-07757-1 (PMC8178906; doi:10.1186/s12864-021-07757-1)
Supplement: Supplementary file 12 — Additional file 12: Figure S10. The average variability in abundance of individual members is greater than the overall variability of the family for most tissues. Scatter plots showing the coefficient of variation of the total abundance of a family across samples of a specific tissue as a function of the mean coefficient of variation of abundance of all members of the family in the tissue. C/D snoRNA families are shown in the graphs in the left and H/ACA snoRNAs are shown on the right. Only expressed family members are considered. The families were colored according to their numbers of expressed members (legend shown at bottom). [file 12864_2021_7757_MOESM12_ESM.pdf]

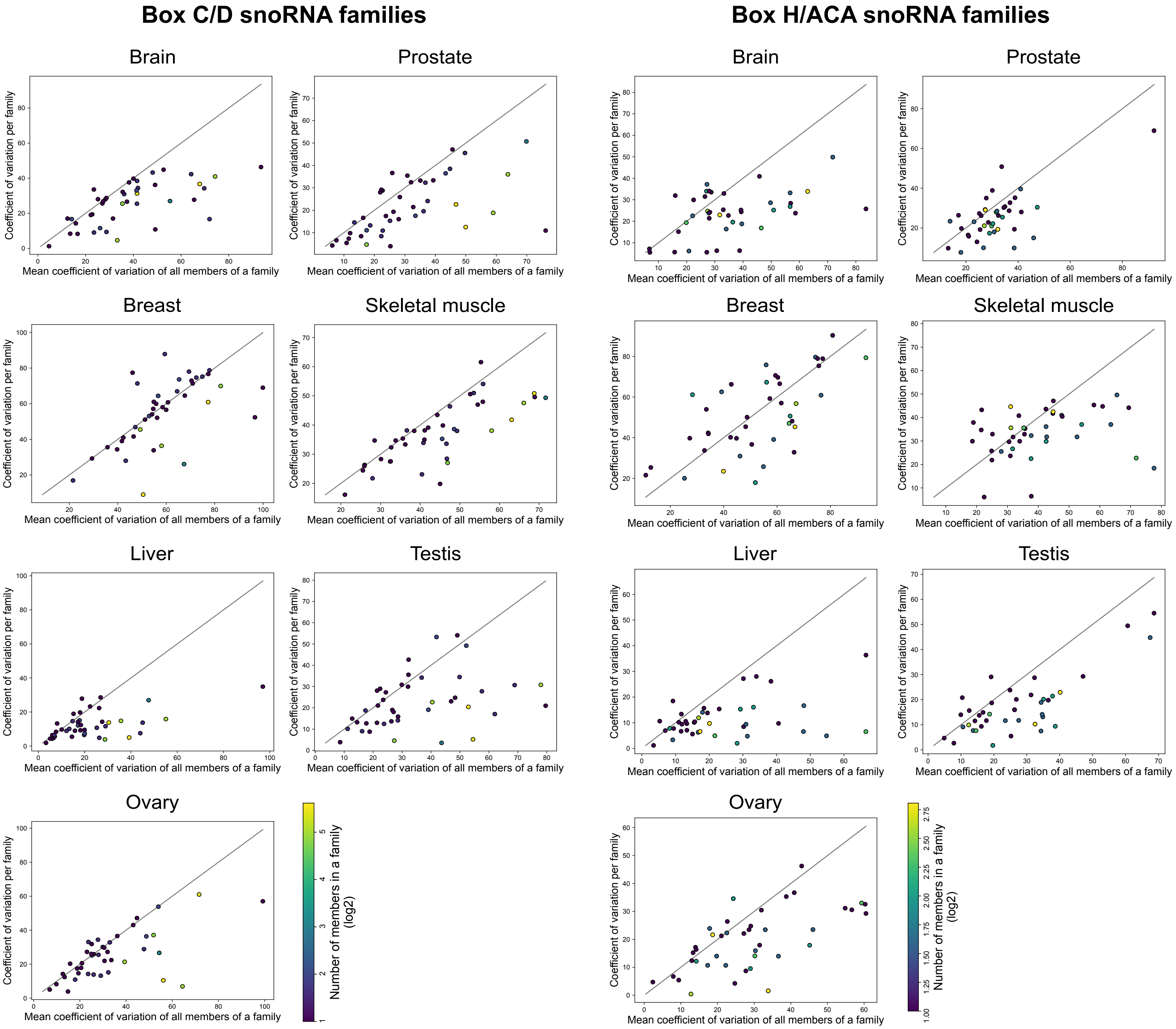

Figure S10: The average variability in abundance of individual members is greater than the overall variability of the family for most tissues. Scatter plots showing the coefficient of variation of the total abundance of a family across samples of a specific tissue as a function of the mean coefficient of variation of abundance of all members of the family in the tissue. C/D snoRNA families are shown in the graphs in the left and H/ACA snoRNAs are shown on the right. Only expressed family members are considered. The families were colored according to their numbers of expressed members (legend shown at bottom).
