# Supplementary material for: SnoRNA copy regulation affects family size, genomic location and family abundance levels
Source: BMC Genomics. 2021 Jun 5;22:414. doi: 10.1186/s12864-021-07757-1 (PMC8178906; doi:10.1186/s12864-021-07757-1)
Supplement: Supplementary file 13 — Additional file 13: Figure S11. Box H/ACA families display higher member variability relative to their total family variability compared to box C/D. Density plot showing the distribution of the distance of each family from the linear function x = y, representing equal total family covariation and mean family members covariation (see Fig. 4B). Negative values represent higher mean family members covariation while positive values represent higher family covariation. Box H/ACA distribution (blue) is significantly different (shifted towards the left) from box C/D distribution (red) according to the Mann-Whitney U test (p-value = 0.042). [file 12864_2021_7757_MOESM13_ESM.pdf]

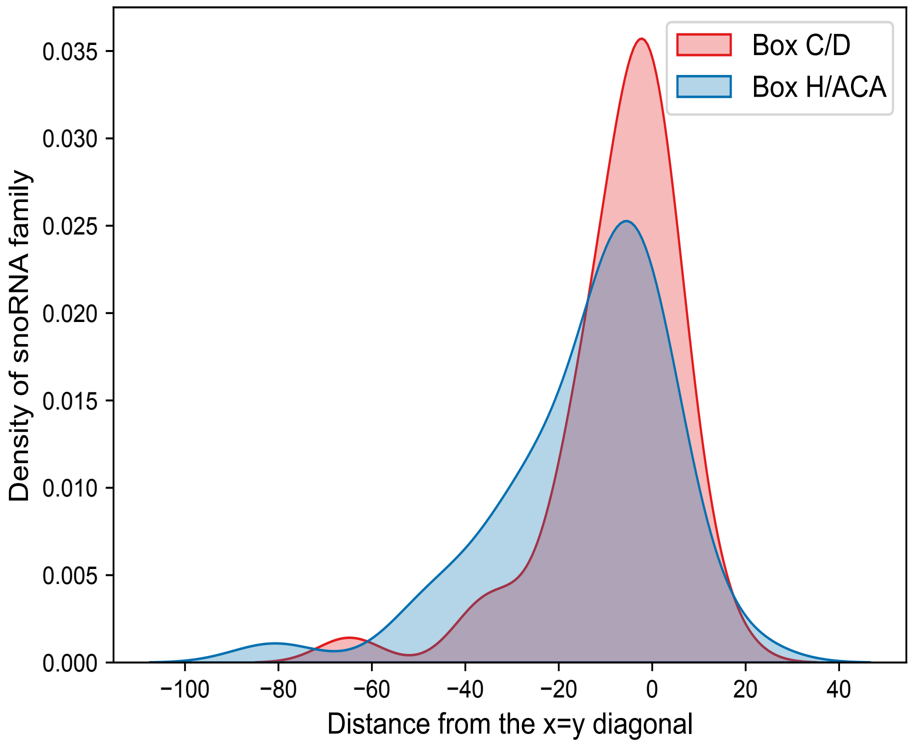

Figure S11: Box H/ACA families display higher member variability relative to their total family variability compared to box C/D. Density plot showing the distribution of the distance of each family from the linear function x=y, representing equal total family covariation and mean family members covariation (see figure 4B). Negative values represent higher mean famliy members covariation while positive values represent higher family covariation. Box H/ACA distribution (blue) is significantly different (shifted towards the left) from box C/D distribution (red) according to the Mann-Whitney U test (p-value=0.042).
