# Supplementary figures and images for: SnoRNA copy regulation affects family size, genomic location and family abundance levels
Source: BMC Genomics. 2021 Jun 5;22:414. doi: 10.1186/s12864-021-07757-1 (PMC8178906; doi:10.1186/s12864-021-07757-1)

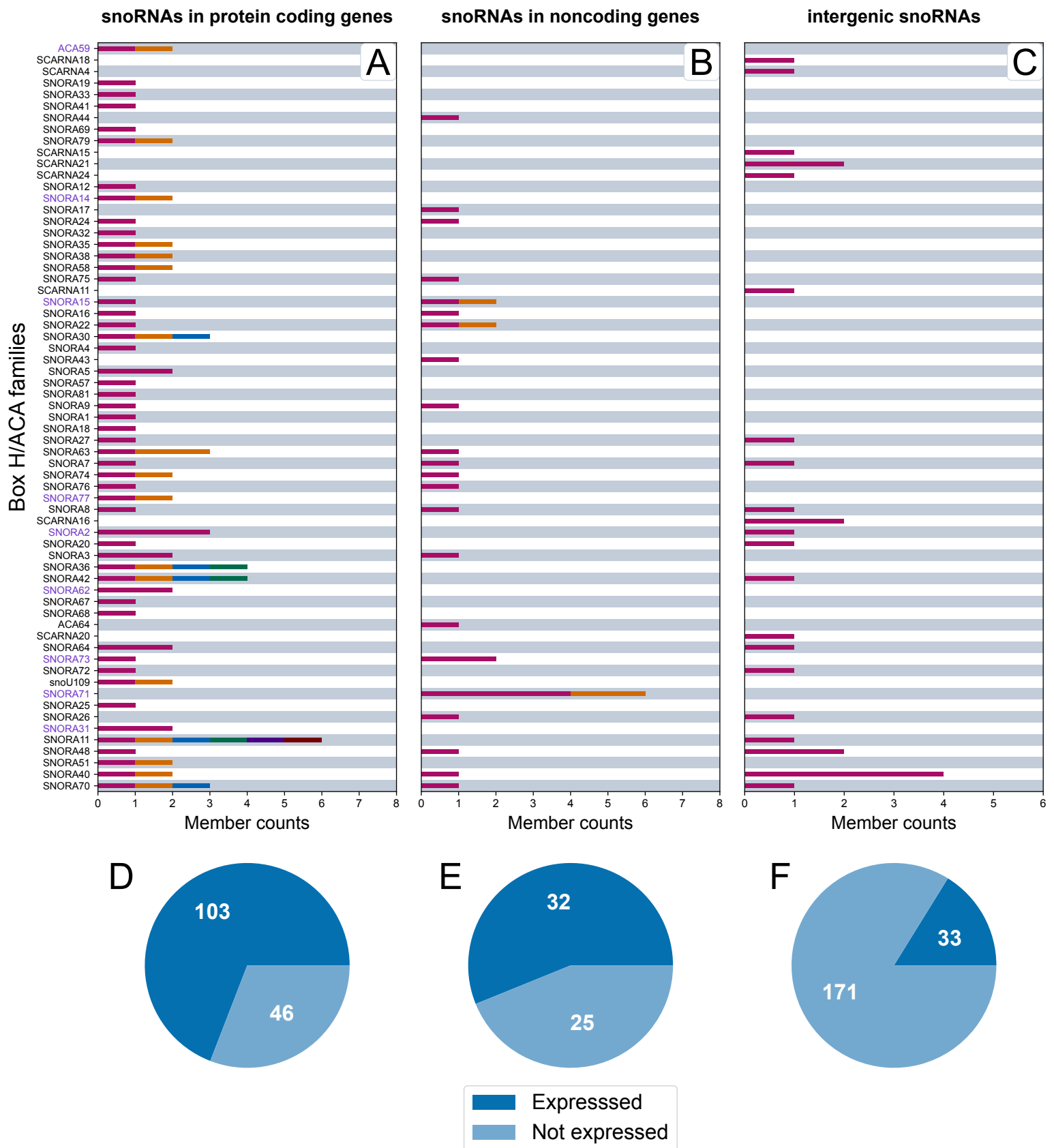

Supplement: Supplementary file 15 — Additional file 15: Figure S13. Human H/ACA snoRNAs are mostly intronic and members can be encoded in more than one distinct host gene. Bar charts displaying the number of intronic (in both coding (A) and noncoding (B) host genes) or intergenic (C) members for box H/ACA families. Only expressed members were shown in the bar charts. In the case of intronic members, the different colors represent different host genes. The proportion of expressed and non-expressed members encoded in intronic coding, intronic noncoding and intergenic regions are shown respectively in D, E and F. The violet in snoRNA family names highlights the presence of a tissue abundance switch between members in the family. [file 12864_2021_7757_MOESM15_ESM.pdf]
