# Supplementary material for: SnoRNA copy regulation affects family size, genomic location and family abundance levels
Source: BMC Genomics. 2021 Jun 5;22:414. doi: 10.1186/s12864-021-07757-1 (PMC8178906; doi:10.1186/s12864-021-07757-1)
Supplement: Supplementary file 16 — Additional file 16: Figure S14. H/ACA snoRNA copies can be regulated in a tissue-specific manner. (A) Differential top tissue for expressed members of a same family. Local scatterplot of each family displaying the abundance of each member of the indicated H/ACA families. The color of the circles represents the tissue in which the member is most abundant. (B) The rank of abundance of members of a family can change between tissues. Categorical heatmap showing the member of highest abundance for all H/ACA families across tissues. Only families with two or more expressed members in at least one tissue are shown. Member 1 is the member with highest total abundance in all tissues, member 2 has the second highest total abundance, and so on. Families in which at least two distinct members are the most abundant in tissues are said to have a switch in the most abundant member across tissues. (C-F) Abundance patterns of family members across tissues. Bar charts displaying the abundance of all expressed (> = 1 average TPM) members across all tissues considered for a given family. Families can display consistent ranking across tissues as shown for the SNORA7 family, or switches between family members as shown for the SNORA71, SNORA14 and SNORA77 families. [file 12864_2021_7757_MOESM16_ESM.pdf]

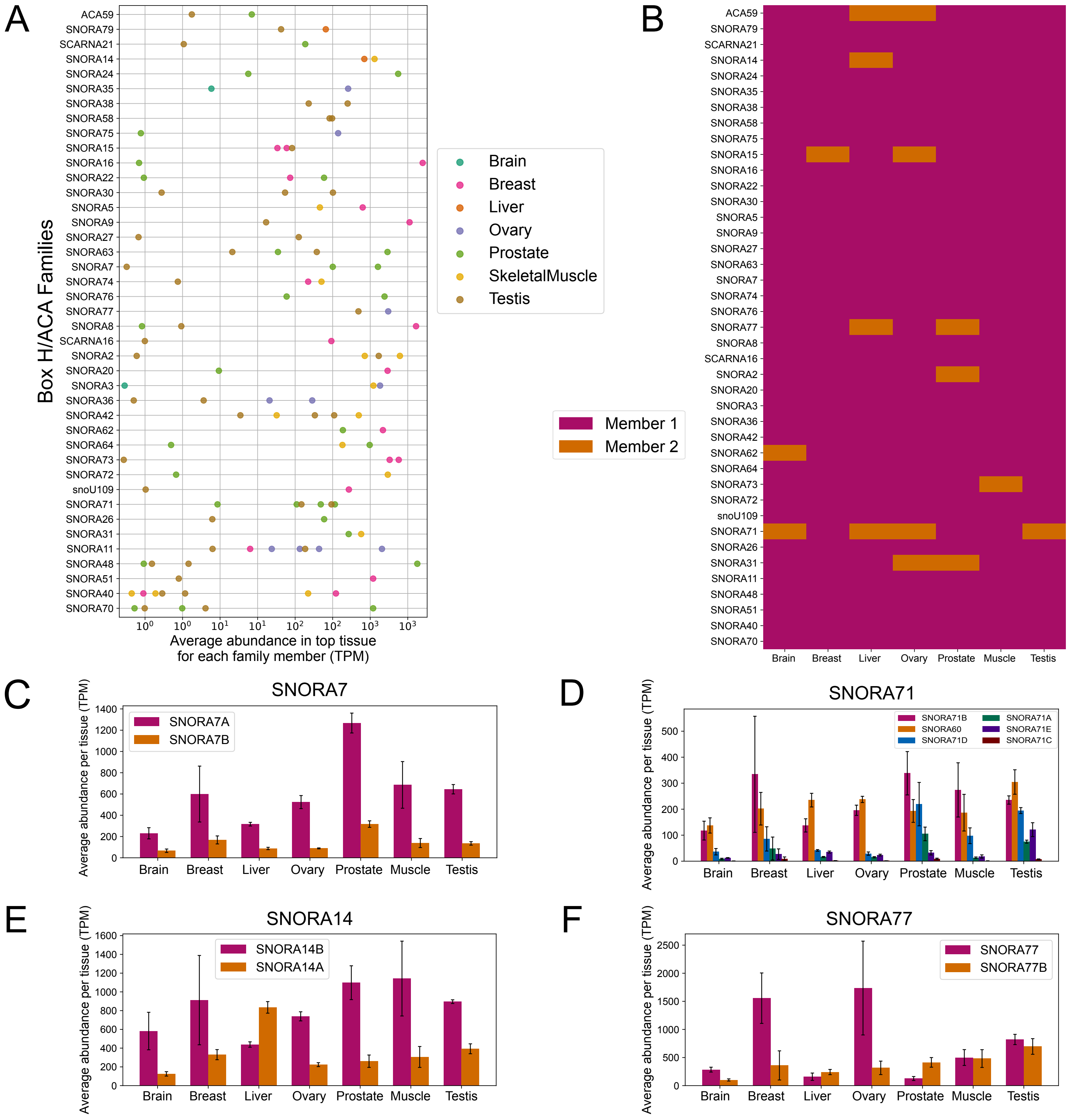

Figure S14: H/ACA snoRNA copies can be regulated in a tissue-specific manner. (A) Differential top tissue for expressed members of a same family. Local scatterplot of each family displaying the abundance of each member of the indicated H/ACA families. The color of the circles represents the tissue in which the member is most abundant. (B) The rank of abundance of members of a family can change between tissues. Categorical heatmap showing the member of highest abundance for all H/ACA families across tissues. Only families with two or more expressed members in at least one tissue are shown. Member 1 is the member with highest total abundance in all tissues, member 2 has the second highest total abundance, and so on. Families in which at least two distinct members are the most abundant in tissues are said to have a switch in the most abundant member across tissues. (C-F) Abundance patterns of family members across tissues. Bar charts displaying the abundance of all expressed (>=1 average TPM) members across all tissues considered for a given family. Families can display consistent ranking across tissues as shown for the SNORA7 family, or switches between family members as shown for the SNORA71, SNORA14 and SNORA77 families.
